# Supplementary material for: Live well, die well – an international cohort study on experiences, concerns and preferences of patients in the last phase of life: the research protocol of the iLIVE study
Source: BMJ Open. 2022 Aug 5;12(8):e057229. doi: 10.1136/bmjopen-2021-057229 (PMC9362824; doi:10.1136/bmjopen-2021-057229)
Supplement: Supplementary data [file bmjopen-2021-057229supp001.pdf]

Supplementary Table 1. The Supportive and Palliative Care Indicators Tool (SPICT).

**General SPICT indicators:**
• Unplanned hospital admission.<br>• Performance status is poor or deteriorating, with limited reversibility (e.g. stays in bed or in a chair for more than half the day).<br>• Depends on others for care due to increasing physical and/or mental health problems; person's carer needs more help and support.<br>• Progressive weight loss; remains underweight; low muscle mass.<br>• Persistent symptoms despite optimal treatment of underlying condition(s).<br>• Person (or family) asks for palliative care; chooses to reduce, stop or not have treatment; or wishes to focus on quality of life.
**Disease-specific SPICT indicators:**
Cancer:<br>• Functional ability deteriorating due to progressive cancer.<br>• Too frail for cancer treatment or treatment is for symptom control.
Neurological disease:<br>• Progressive deterioration in physical and/or cognitive function despite optimal therapy.<br>• Speech problems with increasing difficulty communicating and/or progressive difficulty with swallowing.<br>• Recurrent aspiration pneumonia; breathless or respiratory failure.<br>• Persistent paralysis after stroke with significant loss of function and ongoing disability.
Heart/vascular disease:<br>• Heart failure or extensive, untreatable coronary artery disease; with breathlessness or chest pain at rest or on minimal effort.<br>• Severe, inoperable peripheral vascular disease.
Respiratory disease:<br>• Severe, chronic lung disease; with breathlessness at rest or on minimal effort between exacerbations.<br>• Persistent hypoxia needing long-term oxygen therapy.<br>• Has needed ventilation for respiratory failure or ventilation is contraindicated.
Kidney disease:<br>• Stage 4 or 5 chronic kidney disease (eGFR < 30ml/min) with deteriorating health.<br>• Kidney failure complicating other life limiting conditions or treatments.<br>• Stopping or not starting dialysis.
Liver disease:<br>• Cirrhosis with one or more complications in the past year: diuretic resistant ascites; hepatic encephalopathy; hepatorenal syndrome; bacterial peritonitis; or recurrent variceal bleeds.<br>• Liver transplant is not possible.
Dementia/ frailty[1]:<br>• Unable to dress, walk or eat without help.<br>• Eating and drinking less, difficulty with swallowing.<br>• Urinary and faecal incontinence.<br>• Not able to communicate by speaking; little social interaction.<br>• Frequent falls; fractured femur.<br>• Recurrent febrile episodes or infections, aspiration pneumonia.
Other conditions:<br>• Deteriorating and at risk of dying with other conditions or complications that are not reversible; any treatment available will have a poor outcome.

[1] If a patient with mild cognitive impairment is considered eligible, the physician is requested to assess this patient's capacity using a locally available validated capacity assessment instrument.

Supplementary Table 2. Measurement instruments and their scale scores used in the iLIVE study.

Topic	Measurement instrument	Scale scores
Patients		
- Concerns, expectations and preferences of patients around dying and end-of-life care	- Self-developed questions adapted from the Serious Illness Conversation Guide (1)<br><br>- AEOLI questionnaire (2)	Not applicable<br><br>Strongly disagree – disagree- neither agree nor disagree – agree- strongly agree – don't know
- Symptom load	Edmonton Symptom Assessment System (ESAS) (3)	0 (no symptom) – 10 (worst possible symptom)
- Health-related quality of life (HRQoL) and wellbeing	EORTC QLQ-C15-PAL quality of life question (4)<br><br>EuroQol 5 Dimension questionnaire (EQ-5D-5L) (5)<br><br>ICECAP Supportive Care Measure (ICECAP-SCM) (6)	0 (worst health) – 100 (best health)<br><br>Questions 1-3: no problems – slight problems- moderate problems – severe problems - unable<br><br>Questions 4 (pain) & 5 (anxious): no(t) – slight – moderate – severe – extreme(ly)<br><br>Most of the time –some of the time – only a little of the time - never
- Attitudes towards euthanasia[a]	10-item Euthanasia scale (7)	Strongly disagree – disagree- neither agree nor disagree – agree- strongly agree – don't know
- Health and social care resource use, absenteeism from work	(Partial) Health Economics Questionnaire (HEQ)(8)	Not applicable
- Sociodemographic characteristics	Self-developed questions and HEQ	Not applicable
Relatives		
- Concerns, expectations and preferences around dying and end-of-life care	Self-developed questions inspired by the Serious Illness Conversation Guide and the AEOLI questionnaire	Not applicable

- Health-related quality of life (HRQoL)	EORTC QLQ-C15-PAL<br><br>EQ-5D-5L	0 (worst health) – 100 (best health)<br><br>Questions 1-3: no problems – slight problems- moderate problems – severe problems - unable<br><br>Questions 4 (pain) & 5 (anxious): no(t) – slight – moderate – severe – extreme(ly)
- Well-being	ICECAP Close Person Questionnaire (ICECAP-CPM) (9)	Question 1 -2 : all of the time- most- some- a little- non<br><br>Question 3-6: fully able – mostly able- mostly unable –completely unable
- Informal care provision	iMTA Valuation of Informal Care Questionnaire (iVICQ)(10) and Informal Care Cost Assessment Questionnaire (CIIQ) (11)	Not applicable
- Attitudes towards euthanasia	10-item Euthanasia scale	Strongly disagree – disagree- neither agree nor disagree – agree- strongly agree – don't know
- Bereavement	Hogan Grief Reaction Checklist (HGRC, despair and personal growth subscales) (12)	1= Does not describe me at all<br>2 = Does not quite describe me<br>3 = Describes me fairly well<br>4 = Describes me well<br>5 = Describes me very well
- Quality of care for dying patients	International questionnaire Care of the Dying Evaluation (iCODE) (13)	Various scales
Physicians		
- Patients' diagnosis, co-morbidities and life expectancy, perspective on	Based on the SPICT-criteria and the Australian version of the Karnofsky Performance Status (14)	Not applicable

patients' treatment aims and functional status		
- Evaluation of care in the dying phase	Adapted and based on the Swedish Quality of Dying Registry (15)	Various scales

References

1. Bernacki R, Hutchings M, Vick J, Smith G, Paladino J, Lipsitz S, et al. Development of the Serious Illness Care Program: a randomised controlled trial of a palliative care communication intervention. BMJ Open. 2015;5(10):e009032.
2. Catt S, Blanchard M, Addington-Hall J, Zis M, Blizard B, King M. The development of a questionnaire to assess the attitudes of older people to end-of-life issues (AEOLI). Palliative medicine. 2005;19(5):397-401.
3. Bruera E, Kuehn N, Miller MJ, Selmser P, Macmillan K. The Edmonton Symptom Assessment System (ESAS): a simple method for the assessment of palliative care patients. J Palliat Care. 1991;7(2):6-9.
4. Groenvold M, Petersen MA, Aaronson NK, Arraras JI, Blazeby JM, Bottomley A, et al. The development of the EORTC QLQ-C15-PAL: a shortened questionnaire for cancer patients in palliative care. Eur J Cancer. 2006;42(1):55-64.
5. Euroqol. https://euroqol.org/eq-5d-instruments/eq-5d-5l-about/
6. Bailey C, Kinghorn P, Orlando R, Armour K, Perry R, Jones L, et al. 'The ICECAP-SCM tells you more about what I'm going through': A think-aloud study measuring quality of life among patients receiving supportive and palliative care. Palliative medicine. 2016;30(7):642-52.
7. Wasserman J, Clair JM, Ritchey FJ. A scale to assess attitudes toward euthanasia. OMEGA-Journal of Death and Dying. 2005;51(3):229-37.
8. Simon J, Mayer S. HEQ (Health Economics Questionnaire). Zenodo. 2016.
9. Canaway A, Al-Janabi H, Kinghorn P, Bailey C, Coast J. Development of a measure (ICECAP-Close Person Measure) through qualitative methods to capture the benefits of end-of-life care to those close to the dying for use in economic evaluation. Palliative medicine. 2017;31(1):53-62.
10. Hoefman RJ, Van Exel NJA, Brouwer WBF. iMTA valuation of informal care questionnaire MANUAL. 2013.
11. Landfeldt E, Zethraeus N, Lindgren P. Standardized questionnaire for the measurement, valuation, and estimation of costs of informal care based on the opportunity cost and proxy good method. Applied health economics and health policy. 2019;17(1):15-24.
12. S. Hogan DBGLASN. Development and validation of the Hogan grief reaction checklist. Death studies. 2001;25(1):1-32.
13. Mayland CR, Gerlach C, Sigurdardottir K, Hansen MIT, Leppert W, Stachowiak A, et al. Assessing quality of care for the dying from the bereaved relatives' perspective: Using pre-testing survey methods across seven countries to develop an international outcome measure. Palliat Med. 2019;33(3):357-68.
14. Abernethy AP, Shelby-James T, Fazekas BS, Woods D, Currow DC. The Australia-modified Karnofsky Performance Status (AKPS) scale: a revised scale for contemporary palliative care clinical practice [ISRCTN81117481]. BMC Palliative Care. 2005;4(1):7.
15. Martinsson L, Heedman PA, Lundström S, Fransson G, Axelsson B. Validation study of an end-of-life questionnaire from the Swedish Register of Palliative Care. Acta Oncol. 2011;50(5):642-7.
