# Supplementary material for: Live well, die well – an international cohort study on experiences, concerns and preferences of patients in the last phase of life: the research protocol of the iLIVE study
Source: BMJ Open. 2022 Aug 5;12(8):e057229. doi: 10.1136/bmjopen-2021-057229 (PMC9362824; doi:10.1136/bmjopen-2021-057229)
Supplement: Author's manuscript [file bmjopen-2021-057229.draft_revisions.pdf]

BMJ Open

BMJ Open is committed to open peer review. As part of this commitment we make the peer review history of every article we publish publicly available.

When an article is published we post the peer reviewers' comments and the authors' responses online. We also post the versions of the paper that were used during peer review. These are the versions that the peer review comments apply to.

The versions of the paper that follow are the versions that were submitted during the peer review process. They are not the versions of record or the final published versions. They should not be cited or distributed as the published version of this manuscript.

BMJ Open is an open access journal and the full, final, typeset and author-corrected version of record of the manuscript is available on our site with no access controls, subscription charges or pay-per-view fees (http://bmjopen.bmj.com).

If you have any questions on BMJ Open's open peer review process please email info.bmjopen@bmj.com

LIVE WELL, DIE WELL - AN INTERNATIONAL COHORT STUDY ON EXPERIENCES, CONCERNS AND PREFERENCES OF PATIENTS IN THE LAST PHASE OF LIFE: THE RESEARCH PROTOCOL OF THE iLIVE STUDY

Journal:	*BMJ Open*
Manuscript ID	bmjopen-2021-057229
Article Type:	Protocol
Date Submitted by the Author:	08-Sep-2021

Complete List of Authors:	Yildiz, Berivan; Erasmus MC, University Medical Center Rotterdam, Department of Public Health<br>Allan, Simon; Arohanui Hospice<br>Bakan, Misa; University Clinic of Respiratory and Allergic Diseases Golnik<br>Barnestein-Fonseca, Pilar; CUDECA Foundation; Ibima Institute, Group C08: Pharma economy: Clinical and economic evaluation of medication and Palliative Care<br>Berger, Michael ; Medical University of Vienna, Department of Health Economics, Center for Public Health<br>Boughey, Mark; St Vincent's Hospital Melbourne Pty Ltd, Department of Palliative Care<br>Christen, Andri; Inselspital University Hospital Bern, University Center for Palliative Care, Inselspital University Hospital Bern<br>De Simone, Gustavo; Pallium Latinoamérica<br>Egloff, Martina; Inselspital University Hospital Bern, University Center for Palliative Care, Inselspital University Hospital Bern<br>Ellershaw, John; University of Liverpool, Palliative Care Unit, Institute of Life Course and Medical Sciences<br>Elsten, Eline; Erasmus MC, University Medical Center Rotterdam, Department of Public Health; Erasmus MC, University Medical Center Rotterdam, Department of Medical Oncology, Erasmus MC Cancer Institute<br>Eychmüller, S; University hospital, Inselspital Berne<br>Fischer, Claudia; Medical University of Vienna Center for Public Health, Health Economics<br>Fürst, Carl; Lunds Universitet,<br>Geijteman, Eric C.T. ; Erasmus University Medical Centre, Medical Oncology<br>Goldraij, Gabriel; Hospital Privado Universitario de Córdoba<br>Goossensen, Anne; University of Humanistic Studies, Care and Well-being<br>Halfdanardottir, Svandis Iris ; Landspitali haskolasjukrahus, Palliative Care Unit<br>Faksvåg Haugen, Dagny; Haukeland University Hospital, Regional Centre of Excellence for Palliative Care; University of Bergen, Department of Clinical Medicine K1<br>Hedman, Christel ; Lund University, The Institute for Palliative care at Lund University and Region Skåne; Stiftelsen Stockholms Sjukhem

Hoppe, Tanja; University of Cologne, Faculty of Medicine and University Hospital, Department of Palliative Medicine
Hughes, Rosemary; University of Liverpool, Palliative Care Unit, Institute of Life Course and Medical Sciences
Iversen, Grethe; Haukeland University Hospital, Regional Centre of Excellence for Palliative Care
Joshi, Melanie; University of Cologne, Faculty of Medicine and University Hospital, Department of Palliative Medicine
Kodba-Ceh, Hana; University Clinic of Respiratory and Allergic Diseases Golnik
Korfage, Ida; Erasmus MC, University Medical Center Rotterdam, Public Health
Lunder, Urska; University Clinic of Respiratory and Allergic Diseases Golnik
Lüthi, Nora; Inselspital University Hospital Bern, University Center for Palliative Care
Martín-Roselló, Maria ; CUDECA Foundation; IBIMA Institute , Group CA15: Palliative Care
Mason, Stephen; University of Liverpool, Palliative Care Unit, Institute of Life Course and Medical Sciences
MC Glinchey, Tamsin; University of Liverpool, Palliative Care Unit, Institute of Life Course and Medical Sciences
Montilla, Silvi; University of Buenos Aires, Institute of Medical Research A. Lanari
Rasmussen, Birgit; Lunds Universitet, The Institute for Palliative Care
Ruiz-Torreras, Inmaculada; CUDECA Foundation; Ibima Institute, Group CA15: Palliative Care
Schelin, Maria; Lunds Universitet
Sigurdardottir, Katrin ; Haukeland University Hospital, Regional Centre of Excellence for Palliative Care; Haukeland University Hospital, Specialist Palliative Care Team, Department of Anaesthesia and Surgical Services
Sigurdardottir, Valgerdur; Landspitali haskolasjukrahus, Palliative Care Unit
Simon, Judith; Medical University of Vienna,
Smeding, Ruthmarijke ; University of Liverpool, Palliative Care Unit, Institute of Life Course and Medical Sciences
Solvag, Kjersti; Haukeland University Hospital, Regional Centre of Excellence for Palliative Care
Strupp, Julia; University Hospital Cologne, Department of Palliative Medicine
Tripodoro, Vilma; Pallium Latinoamérica; University of Buenos Aires, Institute of Medical Research A. Lanari
van der Kuy, Hugo; Erasmus MC, Clinical Pharmacy
van der Rijt, Carin; Erasmus Medical Center, Internal Oncology
van Zuylen, Lia; Amsterdam University Medical Centres, Department of Medical Oncology
Veloso, Verónica ; University of Buenos Aires, Institute of Medical Research A. Lanari
Vibora-Martin, Eva; CUDECA Foundation
Voltz, Raymond; Universitat zu Koln Medizinische Fakultat, Department of Palliative Medicine
Zambrano, Sofia; Inselspital University Hospital Bern, University Center for Palliative Care
van der Heide, Agnes; Erasmus MC, University Medical Center Rotterdam, Dept. of Public Health

Keywords: PALLIATIVE CARE, PUBLIC HEALTH, Adult palliative care < PALLIATIVE CARE

SCHOLARONE™
Manuscripts

LIVE WELL, DIE WELL - AN INTERNATIONAL COHORT STUDY ON EXPERIENCES, CONCERNS AND PREFERENCES OF PATIENTS IN THE LAST PHASE OF LIFE: THE RESEARCH PROTOCOL OF THE iLIVE STUDY

Berivan Yildiz[1*], Simon Allan[2], Miša Bakan[3], Pilar Barnestein-Fonseca[4,5], Micheal Berger[6], Mark Boughey[7], Andri Christen[8], Gustavo G. De Simone[9], Martina Egloff[8], John Ellershaw[10], Eline ECM Elsten[13,1], Steffen Eychmüller[8], Claudia Fischer[6], Carl Johan Fürst[11,12], Eric Geijteman[1,13], Gabriel Goldraij[14], Anne Goossensen[15], Svandis Iris Halfdanardottir[16], Dagny Faksvåg Haugen[17,18], Christel Hedman[11,12,19], Tanja Hoppe[20], Rosemary Hughes[10], Grethe Skorpen Iversen[17], Melanie Joshi[20], Hana Kodba-Čeh[3], Ida J. Korfage[1], Urška Lunder[3], Nora Lüthi[8], Maria Luisa Martín-Roselló[4,21], Stephen Mason[10], Tamsin Mc Glinchey[10], S Montilla[22], Birgit H. Rasmussen[11], Inmaculada Ruiz-Torreras[4,21], Maria Schelin[11,12], Katrin Ruth Sigurdardottir[17,23], Valgerdur Sigurdardottir[16], Judit Simon[6], Ruthmarijke Smeding[10], Kjersti Solvåg[17], Julia Strupp[20], Vilma A. Tripodoro[9,22], Hugo van der Kuy[24], Carin CD van der Rijt[13], Lia van Zuylen[25], Verónica I. Veloso[22], Eva Vibora-Martin[4], Raymond Voltz[20,26-28], Sofia C. Zambrano[8], Agnes van der Heide[1].

[1] Erasmus MC, University Medical Center Rotterdam, Department of Public Health, The Netherlands.
[2] Arohanui Hospice, Palmerston North, New Zealand.
[3] University Clinic of Respiratory and Allergic Diseases Golnik, Golnik, Slovenia.
[4] CUDECA Institute for Training and Research in Palliative Care, CUDECA Hospice Foundation.
[5] IBIMA Institute Group C08: Pharma economy: Clinical and economic evaluation of medication and Palliative Care, Málaga, Spain.
[6] Department of Health Economics, Center for Public Health, Medical University of Vienna, Austria.
[7] Department of Palliative Care, St Vincent's Hospital Melbourne, Australia.
[8] University Center for Palliative Care, Inselspital University Hospital Bern, University of Bern, Bern, Switzerland.
[9] Pallium Latinoamérica, Buenos Aires, Argentina.
[10] Palliative Care Unit, Institute of Life Course and Medical Sciences, University of Liverpool, UK.
[11] The Institute for Palliative care at Lund University and Region Skåne, Lund, Sweden.
[12] Division of Oncology and Pathology, Department of Clinical Sciences Lund, Lund University, Lund, Sweden.
[13] Department of Medical Oncology, Erasmus MC Cancer Institute, Erasmus University Medical Center, Rotterdam, The Netherlands.
[14] Hospital Privado Universitario de Córdoba, Córdoba, Argentina
[15] University of Humanistic Studies, Utrecht, The Netherlands
[16] Palliative Care Unit, Landspitali-National University Hospital, Reykjavik, Iceland.
[17] Regional Centre of Excellence for Palliative Care, Western Norway, Haukeland University Hospital, Bergen, Norway.
[18] Department of Clinical Medicine K1, University of Bergen, Bergen, Norway.
[19] Stiftelsen Stockholms Sjukhem, Stockholm, Sweden.

For peer review only - http://bmjopen.bmj.com/site/about/guidelines.xhtml

[20] University of Cologne, Faculty of Medicine and University Hospital, Department of Palliative Medicine, Germany.
[21] IBIMA Institute Group CA15: Palliative Care, Málaga, Spain.
[22] Institute of Medical Research A. Lanari, University of Buenos Aires, Buenos Aires, Argentina.
[23] Specialist Palliative Care Team, Department of Anaesthesia and Surgical Services, Haukeland University Hospital, Bergen, Norway.
[24] Department of Clinical Pharmacy, Erasmus MC, Rotterdam, Zuid-Holland, The Netherlands.
[25] Department of Medical Oncology, Amsterdam University Medical Center, Amsterdam, The Netherlands.
[26] University of Cologne, Faculty of Medicine and University Hospital, Center for Integrated Oncology Aachen Bonn Cologne Dusseldorf (CIO ABCD), Germany.
[27] University of Cologne, Faculty of Medicine and University Hospital, Clinical Trials Center (ZKS), Cologne, Germany.
[28] University of Cologne, Faculty of Medicine and University Hospital, Center for Health Services Research (ZVFK), Cologne, Germany.

***Address of the corresponding author:**
Berivan Yildiz, Erasmus MC, University Medical Center Rotterdam, Department of Public Health, P.O. Box 2040, 3000 CA Rotterdam, The Netherlands.

Email: b.yildiz@erasmusmc.nl

Word count:3148

For peer review

ABSTRACT

Introduction: Adequately addressing the needs of patients at the end of life and their relatives is pivotal in preventing unnecessary suffering and optimizing their quality of life. The purpose of the iLIVE study is to contribute to high-quality personalized care at the end of life in different countries and cultures, by investigating the experiences, concerns, preferences and use of care of terminally ill patients and their families.

Methods and analysis: The iLIVE study is an international cohort study in which patients with an estimated life expectancy of six months or less are followed until they die. In total, 2200 patients will be included in 11 countries, i.e. 200 per country. In addition, one relative per patient is invited to participate. All participants will be asked to fill in a questionnaire, at baseline and after four weeks. If a patient dies within six months of follow-up, the relative will be asked to fill in a post-bereavement questionnaire. Healthcare use in the last week of life will be evaluated as well; healthcare staff who attended the patient will be asked to fill in a brief questionnaire to evaluate the care that was provided. Qualitative interviews will be conducted with patients, relatives and healthcare professionals in all countries to gain more in-depth insights.

Ethics and dissemination: The cohort study has been approved by Ethical Committees and the institutional review boards (IRB's) of participating institutes in all countries. Results will be disseminated through the project website, publications in scientific journals and at conferences. Within the project, there will be a working group focusing on enhancing the engagement of the community at large with the reality of death and dying.

Trial Registration number: NCT04271085

Keywords: End of life, concerns, preferences, quality of care, cohort study, dying patients

For peer review only - http://bmjopen.bmj.com/site/about/guidelines.xhtml

Strengths and limitations of this study

- Due to the international nature of this study, we are able to investigate end-of-life experiences across different cultures and among groups varying by age, gender, illness and care setting.
- This study combines the perspectives of the most relevant stakeholders: patients who are in the last phase of life and their relatives, as well as healthcare staff providing end-of-life care.
- The study population is relatively large which enables to perform subgroup analyses.
- Although patients in the last phase of life and their caregivers have repeatedly reported to appreciate being given the opportunity to participate in research studies, completing a questionnaire about concerns, preferences and expectations concerning the end of life can be uncomfortable.

For peer review only - http://bmjopen.bmj.com/site/about/guidelines.xhtml

INTRODUCTION

Over the past decades, increasing attention has been given to improving care for people in the last phase of life. Literature suggests that most people wish to be free from pain and other symptoms, to be treated with dignity and respect and to maintain a sense of autonomy and control over their last days (1, 2). In addition, many individuals wish to be informed of their limited life expectancy (3). However, there is a substantial amount of variation in the definition of a 'good death'. Preferences for the end of life are dynamic and influenced by individual and multidimensional characteristics, such as age, gender, illness, care setting, financial resources, culture and social relationships (4).

Medical care at the end of life is not optimally addressing the needs and preferences of all patients (5). This is in many cases caused by barriers such as the unpredictable course of a terminal illness, communication difficulties, and the complexity of care needs of dying patients and their families (6). Many terminally ill patients are for instance unable to express their goals and preferences for medical treatment or care, due to physical deterioration or mental incapacity (7, 8). Moreover, since clinicians tend to focus on diagnosis, therapy and cure, the imminence of death is often not openly and timely acknowledged in patients with an advancing chronic illness (9, 10). A recent longitudinal study reported that end-of-life care was discussed between physicians and patients with terminal cancer in less than 20% of cases, and the frequency of these discussions only increased significantly in the last month of life (11). Consequently, patients often receive treatment aimed at prolonging life until a very late stage in their illness trajectory, with a considerable burden for the patient (12). Inadequately addressing the needs of the patients not only deteriorates the quality of patients' last phase of life, but also increases the risk of complicated grief in bereaved family members (13).

So far, studies have mostly explored the perspectives and experiences of communities and physicians regarding factors that are important in end-of-life care (14-16), but the need to include the perspective of patients and their relatives has been acknowledged as well (6, 17). Currently, however, there is a lack of knowledge on what patients in the last phase of life and their relatives consider important (18). The first aim of the iLIVE study is therefore to provide in-depth understanding of the experiences, concerns, expectations and preferences of patients in the last phase of life and their relatives. The second aim is to assess variability in these concerns, expectations and preferences by culture, gender, age, healthcare-related and socio-economic factors. The international character of the iLIVE study provides a framework for unprecedented international comparative insights. A better understanding of needs and outcomes in

For peer review only - http://bmjopen.bmj.com/site/about/guidelines.xhtml

end-of-life care will thus contribute to the development and advancement of policies to support dignified dying in various cultures and settings.

For peer review only

For peer review only - http://bmjopen.bmj.com/site/about/guidelines.xhtml

METHODS AND ANALYSIS

Study design and setting

The iLIVE study is a prospective observational cohort study involving terminally ill patients in hospital and non-hospital sites in 11 participating countries: Argentina, Germany, Iceland, the Netherlands, New Zealand, Norway, Slovenia, Sweden, Switzerland, Spain, and the United Kingdom. Terminally ill patients will be followed until they die or for a maximum of six months after inclusion. Participating patients and one of their relatives will complete questionnaires about their experiences, concerns, expectations and preferences around dying and use of end-of-life care.

The study has been approved by Ethical Committees and institutional review boards (IRB's) in all participating countries. This study is registered in ClinicalTrials.gov (Trial Registration number NCT04271085). A Data Safety Monitoring Board (DSMB) has been established.

Study population

In total, 2200 patients with a maximum estimated life expectancy of six months will be included, regardless of their diagnosis, gender, or place of residence (Table 1). Eligibility is assessed using a modified version of the Gold Standards Framework Proactive Identification Guidance (GSF-PIG) and the Supportive and Palliative Care Indicators Tool (SPICT) (17). The GSF-PIG starts with the "surprise question", asking whether the physician would be surprised if a patient were to die within one year (19). For the present study, we adapted this question into whether the physician would be surprised if a patient were to die within six months. If the physician is uncertain about the surprise question, the patient is eligible when at least one SPICT indicator is present (20). SPICT is a tool to identify persons with poor or deteriorating health for assessment and care planning, using general indicators and clinical signs of life-limiting conditions (Supplemental Table 1).

Participating patients are asked to identify a relative, for instance, a family member or friend. Relatives are eligible if they are 18 years of age or older. Patients and relatives need to be aware that the patient is unlikely to recover from his or her illness. The exclusion criteria for patients also apply to relatives.

For peer review only - http://bmjopen.bmj.com/site/about/guidelines.xhtml

Table 1. Inclusion and exclusion criteria for patients and relatives.

Inclusion criteria for patients	*Exclusion criteria for patients and relatives*
18 years of age or older	Unable to provide informed consent
Attending physician would not be surprised if the patient were to die within 6 months	Incapable of filling in questionnaires in the country's main language or in English
In case of uncertainty about surprise question: at least one SPICT indicator	
Awareness that recovery is unlikely	
Written informed consent to participate	
Inclusion criteria for relatives	
18 years of age or older	
Awareness that recovery of the patient is unlikely	
Written informed consent to participate	

Recruitment procedure

In the 11 countries, across all participating clinical sites, physicians are responsible for screening patients for eligibility. Eligible patients are informed about the study by their attending physician or nurse, who provides them with an information leaflet. If patients are interested in participating, the researcher contacts them, answers their questions, and asks them if they consent to participate. If the patient consents, the researcher asks them to consider whether a close relative might also be willing to participate. After obtaining written informed consent from patients and, if applicable, relatives, they will be asked to fill in the baseline questionnaire.

Measurements

The iLIVE cohort study includes several measurements (Table 2):

1) Questionnaires. Patients, relatives and attending physicians are asked to fill in questionnaires. Patients and relatives will complete questionnaires upon enrolment in the study (baseline assessment) and four weeks later (follow-up 1). For patients who die during the follow-up period of six months, relatives will also complete a questionnaire eight to ten weeks after the death of the patient (follow-up 2). Questionnaires for patients and relatives are administered on paper, online,

For peer review only - http://bmjopen.bmj.com/site/about/guidelines.xhtml

or through telephone or face-to-face interviews. Physicians will complete a paper questionnaire at patient enrolment (baseline assessment) and after the death of a patient (follow-up 2).

Baseline assessment

The baseline questionnaire for patients includes questions on their experiences, concerns, expectations and preferences around dying and end-of-life care. Questions also address health-related quality of life, symptoms, decision-making, social support, and about attitudes towards euthanasia. Finally, questions are asked about health economic aspects, such as patients' employment status, use of healthcare and informal care needs. Relatives will also complete a questionnaire about their experiences, concerns, expectations and preferences around the last phase of life of the patient, their own health-related quality of life, their employment status and their provision of informal care. Attending physicians fill in a questionnaire about patients' diagnosis, co-morbidities, life expectancy, and their perspective on patients' current treatment aims. Where possible, validated measures are used to collect these data. (Table 2)

Follow-up 1

Four weeks after the baseline assessment, patients and relatives are asked to complete a follow-up questionnaire to assess changes as compared to baseline.

Follow-up 2

In case a participating patient dies, participating relatives are after eight to ten weeks asked to fill in a post-bereavement questionnaire, to assess their experience of the last days of life of the deceased patient, their appreciation of the quality of end-of-life care and family support, and their bereavement process. The physician or another healthcare staff member who attended the patient in the dying phase is also asked to complete a questionnaire to evaluate care in the dying phase.

2) Medical file. Healthcare use in the patient's last week of life is assessed using a checklist. Items to be assessed include: place of care, medical complications, medication use, major medical and surgical interventions and care, goals of care statements, resuscitation policy and non-treatment decisions.

For peer review only - http://bmjopen.bmj.com/site/about/guidelines.xhtml

3) Qualitative interviews. More in-depth insight will be obtained in complementary personal interviews. In each country, five patients, five relatives and five healthcare professionals will be interviewed. The same eligibility criteria apply as in the cohort study. Individuals can participate in the interview study, the cohort study, or both. The sample of interviewees will be controlled for age and gender per country, to allow a comparative analysis. The interviews will be semi-structured using a topic guide that is based on Giger-Davidhizar-Haff's model for cultural assessment in end-of-life care (21), the ABCD model (22) and perception of disease questions (23).

Table 2. Measurements among patients, relatives and physicians within the iLIVE project.

**I. Measured by questionnaire**	Measurement instrument
Patients	
- Concerns, expectations and preferences of patients around dying and end-of-life care	Self-developed questions adapted from the Serious Illness Conversation Guide (24) and the AEOLI questionnaire (25)
- Symptom load	Edmonton Symptom Assessment System (ESAS) (26)
- Health-related quality of life (HRQoL) and wellbeing	EORTC QLQ-C15-PAL quality of life question (27) & EuroQol 5 Dimension questionnaire (EQ-5D-5L) (28) ICECAP Supportive Care Measure (ICECAP-SCM) (29)
- Attitudes towards euthanasia[a]	10-item Euthanasia scale (30)
- Health and social care resource use, absenteeism from work	(Partial) Health Economics Questionnaire (HEQ)(31)
- Sociodemographic characteristics	Self-developed questions and HEQ
Relatives	
- Concerns, expectations and preferences around dying and end-of-life care	Self-developed questions inspired by the Serious Illness Conversation Guide and the AEOLI questionnaire
- Health-related quality of life (HRQoL)	EORTC QLQ-C15-PAL & EQ-5D-5L
- Well-being	ICECAP Close Person Questionnaire (ICECAP-CPM) (32)
- Informal care provision	iMTA Valuation of Informal Care Questionnaire (iVICQ)(33) and Informal Care Cost Assessment Questionnaire (CIIQ) (34)
- Attitudes towards euthanasia	10-item Euthanasia scale
- Bereavement	Hogan Grief Reaction Checklist (HGRC, despair and personal growth subscales) (35)
- Quality of care for dying patients	International questionnaire Care of the Dying Evaluation (iCODE) (36)
Physicians	
- Patients' diagnosis, co-morbidities and life expectancy, perspective on patients' treatment aims and functional status	Based on the SPICT-criteria and the Australian version of the Karnofsky Performance Status (37)
- Evaluation of care in the dying phase	Adapted and based on the Swedish Quality of Dying Registry (38)
**II. Obtained from medical files**	

For peer review only - http://bmjopen.bmj.com/site/about/guidelines.xhtml

-	Use of medical interventions, medication and costs of medical care in the last week of life.
-	Patient survival
**III.**	**Obtained from qualitative interviews**
-	In-depth insights into experiences, concerns, expectations and preferences around dying and end-of-life care among patients, relatives and healthcare professionals.

[a] In Norway and Iceland, one self-developed question will be used instead of the 10-item Euthanasia scale. No questions will be asked about euthanasia in Germany.

Data management

This study will be conducted in accordance with the General Data Protection Regulation and national research ethics and privacy guidelines (39). One common data management system will be used to safely process and store data of all participating patients, relatives and physicians across clinical sites and countries. In some countries, participants can choose to directly enter data into this system; in that case, they consent to use of their e-mail address for communication purposes. In all other cases, data are entered anonymously by selected local research assistants, and a study number will be generated to link data of participants with a local communication database.

Sample size

The primary outcomes are measured at baseline and 4 weeks post-inclusion. It is expected that 30% of all patients who complete the baseline assessment will be lost to follow-up, due to death, significant deterioration of health, or other causes. In that case, 70% of patients who complete the baseline measurement will be able to complete the assessment after 4 weeks at follow-up 1. Further, it is expected that 80% of all patients who complete the baseline assessment can be followed until death, whereas the remaining 20% are expected to either survive until the end of the data collection period or become lost to follow-up. Regarding the relatives, it is expected that in case patients who complete the baseline assessment die during follow-up, half of the bereaved relatives (i.e. 40% of all baseline patients), will be willing to complete a post-bereavement questionnaire (follow-up 2). The total cohort would thus include 2200 patients (n=200 per country) at baseline, 1540 patients (n=140 per country) at follow-up assessment 1, and 880 bereaved relatives (n=80 per country).

Analyses

Primary outcomes

The primary outcomes are experiences, concerns, expectations and preferences around dying and end-of-life care of patients in the last phase of life and their relatives, at baseline and after 4 weeks follow-

For peer review only - http://bmjopen.bmj.com/site/about/guidelines.xhtml

up, and will be described in frequencies and narrative descriptions. Descriptive statistics will be used to summarize characteristics of the study participants (age, gender, education, religion, socioeconomic status, marital status, place of residence, quality of life, symptom load) by country and site. Associations with country and patient characteristics will be analysed in a multilevel modelling approach, taking account of clustering effects at country level. All statistical tests will be two-sided and considered significant if $p < 0.05$. Repeated measures analyses of variance will be conducted to assess the development of outcomes between baseline and 4 weeks follow-up. Multivariate Imputation by Chained Equations (MICE) will be used to handle missing data.

Secondary outcomes

Secondary outcomes for patients include symptom load, HRQoL and wellbeing, and attitudes towards physician-assistance in dying. Secondary outcomes for relatives include HRQoL, well-being, informal care provision, attitudes towards physician-assistance in dying and bereavement. The statistical models and methods used to analyse secondary outcomes are similar to those for the primary outcomes. The relationship of the relative to the patient will be taken into account in multivariable models, in addition to the characteristics mentioned for the analysis of the primary outcome.

Health-economic analysis

The outcomes as assessed in this study allow inter alia for a comprehensive assessment of health resource utilization and costs for medication and care, as well as patients' and relatives' quality of life and well-being. The study therefore includes a cost-effectiveness analyses of interventions used in end-of-life care. In addition, a framework for the value assessment of palliative and end-of-life care will be developed (40).

Qualitative interviews

The interviews will be recorded and transcribed verbatim. Data will be thematically analysed in an iterative process on different levels: within each country, within three subgroups of countries and across all countries. The analysis will be focused on identifying experiences, concerns, expectations and preferences, as well as underlying values and norms. In addition, comparison of patients' perspectives will be explored by gender and age to gain a better understanding of differences in phenomena between subgroups.

For peer review only - http://bmjopen.bmj.com/site/about/guidelines.xhtml

Embedded intervention studies

The iLIVE project includes a number of studies that are embedded in the cohort study. The research protocols for these studies will be described elsewhere. A brief description is presented here.

iLIVE medication study

Discussion of appropriate medication to alleviate symptoms is one of the key clinical issues in improving care of dying patients (41). At the same time, potentially inappropriate medication is often continued until a very late stage in patients' illness trajectory (42). This concern will be addressed in the iLIVE Medication Study, in which a digital clinical tool, a so-called Clinical Decision Support System (CDSS), will be used to optimize medication management in the last phase of life. A previous version of this tool to guide physicians in medication prescription and de-prescription for residents of nursing homes was developed and tested in the Netherlands (43). In the iLIVE project, we developed an adapted version of this CDSS, the CDSS-OPTIMED, that supports physicians in optimizing their prescription of medications for patients with a limited life expectancy. The CDSS-OPTIMED will be evaluated in three countries participating in the iLIVE project (The Netherlands, Sweden and Switzerland).

iLIVE volunteer study

Volunteer services to support patients dying in hospitals, and their families, are relatively uncommon and empirical evidence of the usefulness of such services is scarce. This concern will be addressed in the iLIVE Volunteer Study, in which an international hospital palliative care volunteer training programme will be developed . This programme will underpin the implementation of palliative care volunteer services to support patients dying in hospital and their families, within five participating hospitals in five countries (the Netherlands, United Kingdom, Norway, Slovenia, and Spain). The iLIVE Volunteer Study will evaluate the implementation, use and experience of the iLIVE Volunteer Service.

Core Outcome Set for care of the dying

It is important to identify the most important outcomes for care of dying patients through the perspective of patients, family members, researchers, and health professionals. Despite a variety of available tools to assess different dimensions of palliative care, there is no consensus yet on which outcomes need to be measured in the last days of life. Therefore, this project will establish a Core Outcome Set (COS) for care of dying patients that includes valid, reliable and precise outcomes to enable international benchmarking, quality improvement and research in the last days of life (44).

For peer review only - http://bmjopen.bmj.com/site/about/guidelines.xhtml

Patient and Public involvement

The project results will be disseminated through the project website (www.iliveproject.eu), publications in scientific journals and at conferences. Within the project, there will be a working group focusing on enhancing the engagement of the community at large with the reality of death and dying. One of the aims is to actively promote societal debate and engagement with death and dying. This will be achieved by developing a detailed dissemination plan for efficient engagement of citizens, patients and families, healthcare professionals, volunteers and policymakers throughout the project, and effective dissemination of emerging outcomes.

For peer review only

For peer review only - http://bmjopen.bmj.com/site/about/guidelines.xhtml

DISCUSSION

The iLIVE study has several strengths. Going through the last phase of life is a complex personal experience, which is best understood while acknowledging the diverse and dynamic preferences of patients and their families. Due to the international nature of this project, we are able to investigate end-of-life experiences across different cultures and among groups varying by age, gender, illness and care setting. Further, we combine the perspectives of the most relevant stakeholders, that is, patients who are in the last phase of life and their relatives, as well as healthcare staff providing end-of-life care. Furthermore, patients and relatives will complete questionnaires at multiple time points, which enables us to analyse potential adaptations within subjects over time. Another strength relates to the post-bereavement assessment among relatives, which provides insights in the experience of care in the dying phase as well as the impact of these experiences on relatives' wellbeing and bereavement after the death of a patient. Lastly, the study population is relatively large which enables us to perform subgroup analyses.

We expect to encounter several challenges in this study. Recruiting patients in the last phase of life for research studies is often difficult. For instance, healthcare professionals or family members may be hesitant to provide researchers' access to incurably ill patients, due to concerns about burdening or distressing them, a phenomenon referred to as 'gatekeeping' (45). In many studies, this has led to considerably smaller study samples than desired. To minimize this risk, we have involved multiple clinical sites in almost all participating countries, planned for modest numbers of participants per site and applied conservative estimates of expected drop out. In addition, we will screen all potentially eligible patients and keep track of inclusion and exclusion numbers, as well as reasons for non-participation or exclusion.

In addition, completing a questionnaire about concerns, preferences and expectations concerning the end of life can be uncomfortable, especially for patients with a serious illness and a limited life expectancy. However, patients in the last phase of life and their caregivers have repeatedly been reported to appreciate being given the opportunity to participate in research studies, even when they are close to death (46, 47). Participation in this study may nevertheless cause emotional burden for patients, which is why participants are encouraged to indicate this on the questionnaire and contact a

healthcare team member or the research team, so that the patient can be referred for support, e.g. to their GP, attending physician or another healthcare worker. In addition, the study was designed to minimize the data collection burden as much as possible.

Another challenge of this study relates to the COVID-19 pandemic. The COVID-19 pandemic has raised much attention to measures and interventions to protect the health of the population, such as social distancing. Vulnerable people with a severe illness have been particularly affected. Due to COVID-19 associated measures, patients can have an increased risk of unresolved care needs, feelings of abandonment, loneliness, and helplessness, and relatives may, as a result, suffer from prolonged and complicated grief (48). In addition, healthcare professionals have to deal with many pandemic related challenges and may experience significant pressure. It is likely that the experiences and concerns of patients and their relatives will be influenced by measures to control the virus, especially those that are taken during the peaks of the pandemic.

In conclusion, the iLIVE project is aimed at increasing our understanding of the experience of dying in different settings and cultures around the world, and of the concerns, expectations, preferences and needs of dying patients and their relatives. Such understanding is currently lacking, but key to the development of effective and efficient palliative and end-of-life care and public health policies.

Acknowledgements

-

Author contributions

The consortium has evolved from a successful collaboration of clinicians and researchers that developed through a joint interest and expertise in care of the dying to the “International Collaborative for Best Care for the Dying Person”. AVDH, IJD, SA, MB, ME, JE, SE, CF, CJF, EG, AG, SIH, DFH, CH, TH, UL, MLMR, SM, BHR, MS, KRS, VS, JS, RM, KS, JS, VAT, HVDK, CCDVDR, LVZ, RV, SCZ contributed to the design of the study. BY wrote the first draft of the manuscript and revised with input from all authors. All authors read and approved the final manuscript.

For peer review only - http://bmjopen.bmj.com/site/about/guidelines.xhtml

Ethics approval and consent to participate

The cohort study has been approved by Ethical Committees and the institutional review boards (IRB's) of participating institutes in all the countries. Physicians, (research) nurses and research staff will be involved in the recruitment of participants. Patients and their relatives will be asked for permission to be contacted, have the study explained, be invited to participate, be provided written informed consent.

Ethical committees:

- Regional Committees for Medical and Health Research Ethics (35035), Norway.
- Komisija Republike Slovenije za Medicinsko etiko (0120-129/2020/3), Slovenia.
- Health Research Authority (HRA) and Health and Care Research Wales (HCRW) (272927), UK.
- Comité de Ética de la Investigación Provincial de Málaga. Hospital Regional Universitario de Malaga, Spain.
- Swedish Ethical Review Authority (2020-01956). Lund University, Norway.
- The National Bioethics Committee (VSN-20-129), Iceland.
- Ethics Commission of Cologne University, Faculty of Medicine (19-1456_1).
- Gesundheits-, Sozial und Integrationdirektion Kantonale Ethikkommission fur die Forschung (2020-02569), Switzerland.
- Medical research Ethics Committees United (MEC-U) (R20.004), The Netherlands.
- Dictamen del Comité de ética del instituto Lanari, University of Buenos Aires.

Consent to publish

Not applicable.

Competing interests

The authors declare that they have no competing interests.

Funding

This work is supported by the European Union's Horizon 2020 Research and Innovation Programme under Grant agreement no: 825731. The dissemination activities within the iLIVE project do not represent the opinion of the European Community and only reflect the opinion of the authors and/or the Consortium.

Data sharing statement

The study plans to share data per request and as overall study results.

For peer review only - http://bmjopen.bmj.com/site/about/guidelines.xhtml

REFERENCES

1. Heyland DK, Dodek P, Rocker G, Groll D, Gafni A, Pichora D, et al. What matters most in end-of-life care: perceptions of seriously ill patients and their family members. CMAJ. 2006;174(5):627-33.
2. Chochinov HM, Hack T, Hassard T, Kristjanson LJ, McClement S, Harlos M. Dignity in the terminally ill: a cross-sectional, cohort study. Lancet. 2002;360(9350):2026-30.
3. Vig EK, Pearlman RA. Good and bad dying from the perspective of terminally ill men. Arch Intern Med. 2004;164(9):977-81.
4. Thomas C, Morris SM, Clark D. Place of death: preferences among cancer patients and their carers. Soc Sci Med. 2004;58(12):2431-44.
5. Hui D, dos Santos R, Chisholm GB, Bruera E. Symptom Expression in the Last Seven Days of Life Among Cancer Patients Admitted to Acute Palliative Care Units. J Pain Symptom Manage. 2015;50(4):488-94.
6. Kramer B, Auer C. Challenges to Providing End-of-Life Care to Low-Income Elders With Advanced Chronic Disease: Lessons Learned From a Model Program. The Gerontologist. 2005;45:651-60.
7. Silveira MJ, Kim SYH, Langa KM. Advance Directives and Outcomes of Surrogate Decision Making before Death. N Engl J Med. 2010;362(13):1211-8.
8. Heyland DK, Barwich D, Pichora D, Dodek P, Lamontagne F, You JJ, et al. Failure to Engage Hospitalized Elderly Patients and Their Families in Advance Care Planning. JAMA Intern Med. 2013;173(9):778-87.
9. Emanuel EJ, Emanuel LL. The promise of a good death. The Lancet. 1998;351:SII21-SII9.
10. Lee DH. Approach to end of life care. Ochsner J. 2002;4(2):98-103.
11. Tang ST, Chen CH, Wen F-H, Chen J-S, Chang W-C, Hsieh C-H, et al. Accurate Prognostic Awareness Facilitates, Whereas Better Quality of Life and More Anxiety Symptoms Hinder End-of-Life Care Discussions: A Longitudinal Survey Study in Terminally Ill Cancer Patients' Last Six Months of Life. J. Pain Symptom Manag. 2018;55(4):1068-76.
12. Rochigneux P, Raoul JL, Beaussant Y, Aubry R, Goldwasser F, Tournigand C, et al. Use of chemotherapy near the end of life: what factors matter? Ann Oncol. 2017;28(4):809-17.
13. Jonasson JM, Hauksdóttir A, Valdimarsdóttir U, Fürst CJ, Onelöv E, Steineck G. Unrelieved symptoms of female cancer patients during their last months of life and long-term psychological morbidity in their widowers: A nationwide population-based study. Eur J Cancer. 2009;45(10):1839-45.
14. Wong A, Zhukovsky DS. Chapter 3 - Patient Experience and End-of-Life Care: A Discussion and Analysis of Four Patients. In: Gallagher C, Ewer M, editors. Ethical Challenges in Oncology. Boston: Academic Press; 2017. p. 41-57.
15. Black A, McGlinchey T, Gambles M, Ellershaw J, Mayland CR. The 'lived experience' of palliative care patients in one acute hospital setting – a qualitative study. BMC Palliat Care. 2018;17(1):91.
16. Sampson C, Finlay I, Byrne A, Snow V, Nelson A. The practice of palliative care from the perspective of patients and carers. BMJ Supportive & Palliative Care. 2014;4(3):291.
17. www.goldstandardsframework.org.uk/PIG . (Accessed 6 October 2020)
18. Vig EK, Davenport NA, Pearlman RA. Good deaths, bad deaths, and preferences for the end of life: a qualitative study of geriatric outpatients. J Am Geriatr Soc. 2002;50(9):1541-8.
19. White N, Kupeli N, Vickerstaff V, Stone P. How accurate is the 'Surprise Question' at identifying patients at the end of life? A systematic review and meta-analysis. BMC Med. 2017;15(1):139.

For peer review only - http://bmjopen.bmj.com/site/about/guidelines.xhtml

20. Highet G, Crawford D, Murray SA, Boyd K. Development and evaluation of the Supportive and Palliative Care Indicators Tool (SPICT): a mixed-methods study. BMJ Support Palliat Care. 2014;4(3):285-90.
21. Giger JN, Davidhizar R. The Giger and Davidhizar Transcultural Assessment Model. J Transcult Nurs. 2002;13(3):185-8; discussion 200-1.
22. Srivastava R. The ABC (and DE) of cultural competence in clinical care. Ethnicity and Inequalities in Health and Social Care. 2008;1(1):27-33.
23. Price A, Goodwin L, Rayner L, Shaw E, Hansford P, Sykes N, et al. Illness perceptions, adjustment to illness, and depression in a palliative care population. J. Pain Symptom Manag. 2012;43(5):819-32.
24. Bernacki R, Hutchings M, Vick J, Smith G, Paladino J, Lipsitz S, et al. Development of the Serious Illness Care Program: a randomised controlled trial of a palliative care communication intervention. BMJ Open. 2015;5(10):e009032.
25. Catt S, Blanchard M, Addington-Hall J, Zis M, Blizard B, King M. The development of a questionnaire to assess the attitudes of older people to end-of-life issues (AEOLI). Palliat Med. 2005;19(5):397-401.
26. Bruera E, Kuehn N, Miller MJ, Selmser P, Macmillan K. The Edmonton Symptom Assessment System (ESAS): a simple method for the assessment of palliative care patients. J Palliat Care. 1991;7(2):6-9.
27. Groenvold M, Petersen MA, Aaronson NK, Arraras JI, Blazeby JM, Bottomley A, et al. The development of the EORTC QLQ-C15-PAL: a shortened questionnaire for cancer patients in palliative care. Eur J Cancer. 2006;42(1):55-64.
28. Euroqol. https://euroqol.org/eq-5d-instruments/eq-5d-5l-about/
29. Bailey C, Kinghorn P, Orlando R, Armour K, Perry R, Jones L, et al. 'The ICECAP-SCM tells you more about what I'm going through': A think-aloud study measuring quality of life among patients receiving supportive and palliative care. Palliat Med. 2016;30(7):642-52.
30. Wasserman J, Clair JM, Ritchey FJ. A scale to assess attitudes toward euthanasia. OMEGA-Journal of Death and Dying. 2005;51(3):229-37.
31. Simon J, Mayer S. HEQ (Health Economics Questionnaire). Zenodo. 2016.
32. Canaway A, Al-Janabi H, Kinghorn P, Bailey C, Coast J. Development of a measure (ICECAP-Close Person Measure) through qualitative methods to capture the benefits of end-of-life care to those close to the dying for use in economic evaluation. Palliative medicine. 2017;31(1):53-62.
33. Hoefman RJ, Van Exel NJA, Brouwer WBF. iMTA valuation of informal care questionnaire MANUAL. 2013.
34. Landfeldt E, Zethraeus N, Lindgren P. Standardized questionnaire for the measurement, valuation, and estimation of costs of informal care based on the opportunity cost and proxy good method. Appl Health Econ Health Policy. 2019;17(1):15-24.
35. S. Hogan DBGLASN. Development and validation of the Hogan grief reaction checklist. Death studies. 2001;25(1):1-32.
36. Mayland CR, Gerlach C, Sigurdardottir K, Hansen MIT, Leppert W, Stachowiak A, et al. Assessing quality of care for the dying from the bereaved relatives' perspective: Using pre-testing survey methods across seven countries to develop an international outcome measure. Palliat Med. 2019;33(3):357-68.
37. Abernethy AP, Shelby-James T, Fazekas BS, Woods D, Currow DC. The Australia-modified Karnofsky Performance Status (AKPS) scale: a revised scale for contemporary palliative care clinical practice [ISRCTN81117481]. BMC Palliat. Care. 2005;4(1):7.
38. Martinsson L, Heedman PA, Lundström S, Fransson G, Axelsson B. Validation study of an end-of-life questionnaire from the Swedish Register of Palliative Care. Acta Oncol. 2011;50(5):642-7.
39. https://www.eugdpr.org/.

For peer review only - http://bmjopen.bmj.com/site/about/guidelines.xhtml

40. Fischer C, Chwala E, Simon J. Methodological aspects of economic evaluations conducted in the palliative or end of life care settings: a systematic review protocol. BMJ Open. 2020;10(5):e035760.
41. Todd A, Holmes HM. Recommendations to support deprescribing medications late in life. Intern J. Clin Pharm. 2015;37(5):678-81.
42. Arevalo JJ, Geijteman ECT, Huisman BAA, Dees MK, Zuurmond WWA, van Zuylen L, et al. Medication Use in the Last Days of Life in Hospital, Hospice, and Home Settings in the Netherlands. J Palliat Med. 2018;21(2):149-55.
43. Mestres Gonzalvo C, de Wit HAJM, van Oijen BPC, Hurkens KPGM, Janknegt R, Schols JMGA, et al. Supporting clinical rules engine in the adjustment of medication (SCREAM): protocol of a multicentre, prospective, randomised study. BMC Geriatr. 2017;17(1):35-.
44. Zambrano SC, Haugen DF, van der Heide A, Tripodoro VA, Ellershaw J, Fürst CJ, et al. Development of an international Core Outcome Set (COS) for best care for the dying person: study protocol. BMC Palliat Care. 2020;19(1):184.
45. Kars MC, van Thiel GJ, van der Graaf R, Moors M, de Graeff A, van Delden JJ. A systematic review of reasons for gatekeeping in palliative care research. Palliat Med. 2016;30(6):533-48.
46. Gysels MH, Evans C, Higginson IJ. Patient, caregiver, health professional and researcher views and experiences of participating in research at the end of life: a critical interpretive synthesis of the literature. BMC Med Res Methodol. 2012;12:123.
47. Higginson IJ, Evans CJ, Grande G, Preston N, Morgan M, McCrone P, et al. Evaluating complex interventions in end of life care: the MORECare statement on good practice generated by a synthesis of transparent expert consultations and systematic reviews. BMC Med. 2013;11(1):111.
48. Strang P, Bergström J, Martinsson L, Lundström S. Dying From COVID-19: Loneliness, End-of-Life Discussions, and Support for Patients and Their Families in Nursing Homes and Hospitals. A National Register Study. J. Pain Symptom Manag. 2020;60(4):e2-e13.

For peer review only

For peer review only - http://bmjopen.bmj.com/site/about/guidelines.xhtml

Supplementary Table 1. The Supportive and Palliative Care Indicators Tool (SPICT).

**General SPICT indicators:**
• Unplanned hospital admission.<br>• Performance status is poor or deteriorating, with limited reversibility (e.g. stays in bed or in a chair for more than half the day).<br>• Depends on others for care due to increasing physical and/or mental health problems; person's carer needs more help and support.<br>• Progressive weight loss; remains underweight; low muscle mass.<br>• Persistent symptoms despite optimal treatment of underlying condition(s).<br>• Person (or family) asks for palliative care; chooses to reduce, stop or not have treatment; or wishes to focus on quality of life.
**Disease-specific SPICT indicators:**
Cancer:<br>• Functional ability deteriorating due to progressive cancer.<br>• Too frail for cancer treatment or treatment is for symptom control.
Neurological disease:<br>• Progressive deterioration in physical and/or cognitive function despite optimal therapy.<br>• Speech problems with increasing difficulty communicating and/or progressive difficulty with swallowing.<br>• Recurrent aspiration pneumonia; breathless or respiratory failure.<br>• Persistent paralysis after stroke with significant loss of function and ongoing disability.
Heart/vascular disease:<br>• Heart failure or extensive, untreatable coronary artery disease; with breathlessness or chest pain at rest or on minimal effort.<br>• Severe, inoperable peripheral vascular disease.
Respiratory disease:<br>• Severe, chronic lung disease; with breathlessness at rest or on minimal effort between exacerbations.<br>• Persistent hypoxia needing long-term oxygen therapy.<br>• Has needed ventilation for respiratory failure or ventilation is contraindicated.
Kidney disease:<br>• Stage 4 or 5 chronic kidney disease (eGFR < 30ml/min) with deteriorating health.<br>• Kidney failure complicating other life limiting conditions or treatments.<br>• Stopping or not starting dialysis.
Liver disease:<br>• Cirrhosis with one or more complications in the past year: diuretic resistant ascites; hepatic encephalopathy; hepatorenal syndrome; bacterial peritonitis; or recurrent variceal bleeds.<br>• Liver transplant is not possible.
Dementia/ frailty[1]:<br>• Unable to dress, walk or eat without help.<br>• Eating and drinking less, difficulty with swallowing.<br>• Urinary and faecal incontinence.<br>• Not able to communicate by speaking; little social interaction.<br>• Frequent falls; fractured femur.<br>• Recurrent febrile episodes or infections, aspiration pneumonia.
Other conditions:<br>• Deteriorating and at risk of dying with other conditions or complications that are not reversible; any treatment available will have a poor outcome.

[1] If a patient with mild cognitive impairment is considered eligible, the physician is requested to assess this patient's capacity using a locally available validated capacity assessment instrument.

For peer review only

For peer review only

STROBE 2007 (v4) Statement—Checklist of items that should be included in reports of *cohort studies*

Section/Topic	Item #	Recommendation	Reported on page #
**Title and abstract**	1	(*a*) Indicate the study's design with a commonly used term in the title or the abstract	0
		(*b*) Provide in the abstract an informative and balanced summary of what was done and what was found	1
**Introduction**			
Background/rationale	2	Explain the scientific background and rationale for the investigation being reported	4
Objectives	3	State specific objectives, including any prespecified hypotheses	4
**Methods**			
Study design	4	Present key elements of study design early in the paper	6
Setting	5	Describe the setting, locations, and relevant dates, including periods of recruitment, exposure, follow-up, and data collection	6
Participants	6	(*a*) Give the eligibility criteria, and the sources and methods of selection of participants. Describe methods of follow-up	6/7
		(*b*) For matched studies, give matching criteria and number of exposed and unexposed	na
Variables	7	Clearly define all outcomes, exposures, predictors, potential confounders, and effect modifiers. Give diagnostic criteria, if applicable	7-9
Data sources/ measurement	8*	For each variable of interest, give sources of data and details of methods of assessment (measurement). Describe comparability of assessment methods if there is more than one group	*8-9*
Bias	9	Describe any efforts to address potential sources of bias	na
Study size	10	Explain how the study size was arrived at	10
Quantitative variables	11	Explain how quantitative variables were handled in the analyses. If applicable, describe which groupings were chosen and why	na
Statistical methods	12	(*a*) Describe all statistical methods, including those used to control for confounding	11
		(*b*) Describe any methods used to examine subgroups and interactions	11
		(*c*) Explain how missing data were addressed	11
		(*d*) If applicable, explain how loss to follow-up was addressed	11
		(*e*) Describe any sensitivity analyses	na
**Results**			

For peer review only

Participants	13*	(a) Report numbers of individuals at each stage of study—eg numbers potentially eligible, examined for eligibility, confirmed eligible, included in the study, completing follow-up, and analysed	na
		(b) Give reasons for non-participation at each stage	na
		(c) Consider use of a flow diagram	na
Descriptive data	14*	(a) Give characteristics of study participants (eg demographic, clinical, social) and information on exposures and potential confounders	na
		(b) Indicate number of participants with missing data for each variable of interest	na
		(c) Summarise follow-up time (eg, average and total amount)	na
Outcome data	15*	Report numbers of outcome events or summary measures over time	na
Main results	16	(*a*) Give unadjusted estimates and, if applicable, confounder-adjusted estimates and their precision (eg, 95% confidence interval). Make clear which confounders were adjusted for and why they were included	na
		(*b*) Report category boundaries when continuous variables were categorized	na
		(*c*) If relevant, consider translating estimates of relative risk into absolute risk for a meaningful time period	na
Other analyses	17	Report other analyses done—eg analyses of subgroups and interactions, and sensitivity analyses	na
**Discussion**			
Key results	18	Summarise key results with reference to study objectives	na
**Limitations**			
Interpretation	20	Give a cautious overall interpretation of results considering objectives, limitations, multiplicity of analyses, results from similar studies, and other relevant evidence	na
Generalisability	21	Discuss the generalisability (external validity) of the study results	14
**Other information**			
Funding	22	Give the source of funding and the role of the funders for the present study and, if applicable, for the original study on which the present article is based	16

*Give information separately for cases and controls in case-control studies and, if applicable, for exposed and unexposed groups in cohort and cross-sectional studies.

Note: An Explanation and Elaboration article discusses each checklist item and gives methodological background and published examples of transparent reporting. The STROBE checklist is best used in conjunction with this article (freely available on the Web sites of PLoS Medicine at http://www.plosmedicine.org/, Annals of Internal Medicine at http://www.annals.org/, and Epidemiology at http://www.epidem.com/). Information on the STROBE Initiative is available at www.strobe-statement.org.

For peer review only

BMJ Open

LIVE WELL, DIE WELL - AN INTERNATIONAL COHORT STUDY ON EXPERIENCES, CONCERNS AND PREFERENCES OF PATIENTS IN THE LAST PHASE OF LIFE: THE RESEARCH PROTOCOL OF THE iLIVE STUDY

Journal:	*BMJ Open*
Manuscript ID	bmjopen-2021-057229.R1
Article Type:	Protocol
Date Submitted by the Author:	20-Apr-2022

Complete List of Authors:	Yildiz, Berivan; Erasmus MC, University Medical Center Rotterdam, Department of Public Health<br>Allan, Simon; Arohanui Hospice<br>Bakan, Misa; University Clinic of Respiratory and Allergic Diseases Golnik<br>Barnestein-Fonseca, Pilar; CUDECA Foundation; Ibima Institute, Group C08: Pharma economy: Clinical and economic evaluation of medication and Palliative Care<br>Berger, Michael ; Medical University of Vienna, Department of Health Economics, Center for Public Health<br>Boughey, Mark; St Vincent's Hospital Melbourne Pty Ltd, Department of Palliative Care<br>Christen, Andri; Inselspital University Hospital Bern, University Center for Palliative Care, Inselspital University Hospital Bern<br>De Simone, Gustavo; Pallium Latinoamérica<br>Egloff, Martina; Inselspital University Hospital Bern, University Center for Palliative Care, Inselspital University Hospital Bern<br>Ellershaw, John; University of Liverpool, Palliative Care Unit, Institute of Life Course and Medical Sciences<br>Elsten, Eline; Erasmus MC, University Medical Center Rotterdam, Department of Public Health; Erasmus MC, University Medical Center Rotterdam, Department of Medical Oncology, Erasmus MC Cancer Institute<br>Eychmüller, S; Inselspital University Hospital Bern, University Center for Palliative Care<br>Fischer, Claudia; Medical University of Vienna Center for Public Health, Health Economics<br>Fürst, Carl; Lunds Universitet,<br>Geijteman, Eric C.T. ; Erasmus University Medical Centre, Medical Oncology<br>Goldraij, Gabriel; Hospital Privado Universitario de Córdoba<br>Goossensen, Anne; University of Humanistic Studies, Care and Well-being<br>Halfdanardottir, Svandis Iris ; Landspitali haskolasjukrahus, Palliative Care Unit<br>Faksvåg Haugen, Dagny; Haukeland University Hospital, Regional Centre of Excellence for Palliative Care; University of Bergen, Department of Clinical Medicine K1<br>Hedman, Christel ; Lund University, The Institute for Palliative care at

	Lund University and Region Skåne; Stiftelsen Stockholms Sjukhem<br>Hoppe, Tanja; University of Cologne, Faculty of Medicine and University Hospital, Department of Palliative Medicine<br>Hughes, Rosemary; University of Liverpool, Palliative Care Unit, Institute of Life Course and Medical Sciences<br>Iversen, Grethe; Haukeland University Hospital, Regional Centre of Excellence for Palliative Care<br>Joshi, Melanie; University of Cologne, Faculty of Medicine and University Hospital, Department of Palliative Medicine<br>Kodba-Ceh, Hana; University Clinic of Respiratory and Allergic Diseases Golnik<br>Korfage, Ida; Erasmus MC, University Medical Center Rotterdam, Public Health<br>Lunder, Urska; University Clinic of Respiratory and Allergic Diseases Golnik<br>Lüthi, Nora; Inselspital University Hospital Bern, University Center for Palliative Care<br>Martín-Roselló, Maria ; CUDECA Foundation; IBIMA Institute , Group CA15: Palliative Care<br>Mason, Stephen; University of Liverpool, Palliative Care Unit, Institute of Life Course and Medical Sciences<br>MC Glinchey, Tamsin; University of Liverpool, Palliative Care Unit, Institute of Life Course and Medical Sciences<br>Montilla, Silvi; University of Buenos Aires, Institute of Medical Research A. Lanari<br>Rasmussen, Birgit; Lunds Universitet, The Institute for Palliative Care<br>Ruiz-Torreras, Inmaculada; CUDECA Foundation; Ibima Institute, Group CA15: Palliative Care<br>Schelin, Maria; Lunds Universitet<br>Sigurdardottir, Katrin ; Haukeland University Hospital, Regional Centre of Excellence for Palliative Care; Haukeland University Hospital, Specialist Palliative Care Team, Department of Anaesthesia and Surgical Services<br>Sigurdardottir, Valgerdur; Landspitali haskolasjukrahus, Palliative Care Unit<br>Simon, Judith; Medical University of Vienna,<br>Smeding, Ruthmarijke ; University of Liverpool, Palliative Care Unit, Institute of Life Course and Medical Sciences<br>Solvag, Kjersti; Haukeland University Hospital, Regional Centre of Excellence for Palliative Care<br>Strupp, Julia; University Hospital Cologne, Department of Palliative Medicine<br>Tripodoro, Vilma; Pallium Latinoamérica; University of Buenos Aires, Institute of Medical Research A. Lanari<br>van der Kuy, Hugo; Erasmus MC, Clinical Pharmacy<br>van der Rijt, Carin; Erasmus Medical Center, Internal Oncology<br>van Zuylen, Lia; Amsterdam University Medical Centres, Department of Medical Oncology<br>Veloso, Verónica ; University of Buenos Aires, Institute of Medical Research A. Lanari<br>Vibora-Martin, Eva; CUDECA Foundation<br>Voltz, Raymond; Universitat zu Koln Medizinische Fakultat, Department of Palliative Medicine<br>Zambrano, Sofia; Inselspital University Hospital Bern, University Center for Palliative Care<br>van der Heide, Agnes; Erasmus MC, University Medical Center Rotterdam, Dept. of Public Health
**Primary Subject Heading**:	Palliative care
Secondary Subject Heading:	Epidemiology

Keywords:	PALLIATIVE CARE, PUBLIC HEALTH, Adult palliative care < PALLIATIVE CARE

SCHOLARONE™
Manuscripts

LIVE WELL, DIE WELL - AN INTERNATIONAL COHORT STUDY ON EXPERIENCES, CONCERNS AND PREFERENCES OF PATIENTS IN THE LAST PHASE OF LIFE: THE RESEARCH PROTOCOL OF THE iLIVE STUDY

Berivan Yildiz[1*], Simon Allan[2], Miša Bakan[3], Pilar Barnestein-Fonseca[4,5], Micheal Berger[6], Mark Boughey[7], Andri Christen[8], Gustavo G. De Simone[9], Martina Egloff[8], John Ellershaw[10], Eline ECM Elsten[13,1], Steffen Eychmüller[8], Claudia Fischer[6], Carl Johan Fürst[11,12], Eric Geijteman[1,13], Gabriel Goldraij[14], Anne Goossensen[15], Svandis Iris Halfdanardottir[16], Dagny Faksvåg Haugen[17,18], Christel Hedman[11,12,19], Tanja Hoppe[20], Rosemary Hughes[10], Grethe Skorpen Iversen[17], Melanie Joshi[20], Hana Kodba-Čeh[3], Ida J. Korfage[1], Urška Lunder[3], Nora Lüthi[8], Maria Luisa Martín-Roselló[4,21], Stephen Mason[10], Tamsin Mc Glinchey[10], S Montilla[22], Birgit H. Rasmussen[11], Inmaculada Ruiz-Torreras[4,21], Maria Schelin[11,12], Katrin Ruth Sigurdardottir[17,23], Valgerdur Sigurdardottir[16], Judit Simon[6], Ruthmarijke Smeding[10], Kjersti Solvåg[17], Julia Strupp[20], Vilma A. Tripodoro[9,22], Hugo van der Kuy[24], Carin CD van der Rijt[13], Lia van Zuylen[25], Verónica I. Veloso[22], Eva Vibora-Martin[4], Raymond Voltz[20,26-28], Sofia C. Zambrano[8], Agnes van der Heide[1].

[1] Erasmus MC, University Medical Center Rotterdam, Department of Public Health, The Netherlands.
[2] Arohanui Hospice, Palmerston North, New Zealand.
[3] University Clinic of Respiratory and Allergic Diseases Golnik, Golnik, Slovenia.
[4] CUDECA Institute for Training and Research in Palliative Care, CUDECA Hospice Foundation.
[5] IBIMA Institute Group C08: Pharma economy: Clinical and economic evaluation of medication and Palliative Care, Málaga, Spain.
[6] Department of Health Economics, Center for Public Health, Medical University of Vienna, Austria.
[7] Department of Palliative Care, St Vincent's Hospital Melbourne, Australia.
[8] University Center for Palliative Care, Inselspital University Hospital Bern, University of Bern, Bern, Switzerland.
[9] Pallium Latinoamérica, Buenos Aires, Argentina.
[10] Palliative Care Unit, Institute of Life Course and Medical Sciences, University of Liverpool, UK.
[11] The Institute for Palliative care at Lund University and Region Skåne, Lund, Sweden.
[12] Division of Oncology and Pathology, Department of Clinical Sciences Lund, Lund University, Lund, Sweden.
[13] Department of Medical Oncology, Erasmus MC Cancer Institute, Erasmus University Medical Center, Rotterdam, The Netherlands.
[14] Hospital Privado Universitario de Córdoba, Córdoba, Argentina
[15] University of Humanistic Studies, Utrecht, The Netherlands
[16] Palliative Care Unit, Landspitali-National University Hospital, Reykjavik, Iceland.
[17] Regional Centre of Excellence for Palliative Care, Western Norway, Haukeland University Hospital, Bergen, Norway.
[18] Department of Clinical Medicine K1, University of Bergen, Bergen, Norway.
[19] Stiftelsen Stockholms Sjukhem, Stockholm, Sweden.

For peer review only - http://bmjopen.bmj.com/site/about/guidelines.xhtml

[20] University of Cologne, Faculty of Medicine and University Hospital, Department of Palliative Medicine, Germany.
[21] IBIMA Institute Group CA15: Palliative Care, Málaga, Spain.
[22] Institute of Medical Research A. Lanari, University of Buenos Aires, Buenos Aires, Argentina.
[23] Specialist Palliative Care Team, Department of Anaesthesia and Surgical Services, Haukeland University Hospital, Bergen, Norway.
[24] Department of Clinical Pharmacy, Erasmus MC, Rotterdam, Zuid-Holland, The Netherlands.
[25] Department of Medical Oncology, Amsterdam University Medical Center, Amsterdam, The Netherlands.
[26] University of Cologne, Faculty of Medicine and University Hospital, Center for Integrated Oncology Aachen Bonn Cologne Dusseldorf (CIO ABCD), Germany.
[27] University of Cologne, Faculty of Medicine and University Hospital, Clinical Trials Center (ZKS), Cologne, Germany.
[28] University of Cologne, Faculty of Medicine and University Hospital, Center for Health Services Research (ZVFK), Cologne, Germany.

***Address of the corresponding author:**
Berivan Yildiz, Erasmus MC, University Medical Center Rotterdam, Department of Public Health, P.O. Box 2040, 3000 CA Rotterdam, The Netherlands.
Email: b.yildiz@erasmusmc.nl

Word count: 4324

For peer review only - http://bmjopen.bmj.com/site/about/guidelines.xhtml

ABSTRACT

Introduction: Adequately addressing the needs of patients at the end of life and their relatives is pivotal in preventing unnecessary suffering and optimizing their quality of life. The purpose of the iLIVE study is to contribute to high-quality personalized care at the end of life in different countries and cultures, by investigating the experiences, concerns, preferences and use of care of terminally ill patients and their families.

Methods and analysis: The iLIVE study is an international cohort study in which patients with an estimated life expectancy of six months or less are followed until they die. In total, 2200 patients will be included in 11 countries, i.e. 200 per country. In addition, one relative per patient is invited to participate. All participants will be asked to fill in a questionnaire, at baseline and after four weeks. If a patient dies within six months of follow-up, the relative will be asked to fill in a post-bereavement questionnaire. Healthcare use in the last week of life will be evaluated as well; healthcare staff who attended the patient will be asked to fill in a brief questionnaire to evaluate the care that was provided. Qualitative interviews will be conducted with patients, relatives and healthcare professionals in all countries to gain more in-depth insights.

Ethics and dissemination: The cohort study has been approved by Ethical Committees and the institutional review boards (IRB's) of participating institutes in all countries. Results will be disseminated through the project website, publications in scientific journals and at conferences. Within the project, there will be a working group focusing on enhancing the engagement of the community at large with the reality of death and dying.

Trial Registration number: NCT04271085

Keywords: End of life, concerns, preferences, quality of care, cohort study, dying patients

For peer review only - http://bmjopen.bmj.com/site/about/guidelines.xhtml

Strengths and limitations of this study

- Due to the international nature of this study, we are able to investigate end-of-life experiences across different cultures and among groups varying by age, gender, illness and care setting.

- This study combines the perspectives of the most relevant stakeholders: patients who are in the last phase of life and their relatives, as well as healthcare staff providing end-of-life care.

- The study population is relatively large which enables to perform subgroup analyses.

- Although patients in the last phase of life and their caregivers have repeatedly reported to appreciate being given the opportunity to participate in research studies, completing a questionnaire about concerns, preferences and expectations concerning the end of life can be uncomfortable.

For peer review only - http://bmjopen.bmj.com/site/about/guidelines.xhtml

INTRODUCTION

Over the past decades, increasing attention has been given to improving care for people in the last phase of life. Literature suggests that most people wish to be free from pain and other symptoms, to be treated with dignity and respect and to maintain a sense of autonomy and control over their last days (1-4). In addition, many individuals wish to be informed of their limited life expectancy (5). However, there is a substantial amount of variation in the definition of a 'good death'. Preferences for the end of life are dynamic and influenced by individual and multidimensional characteristics, such as age, gender, illness, care setting, financial resources, culture and social relationships (6).

Medical care at the end of life is not optimally addressing the needs and preferences of all patients (7). This is in many cases caused by barriers such as the unpredictable course of a terminal illness, communication difficulties, and the complexity of care needs of dying patients and their families (8). Many terminally ill patients are for instance unable to express their goals and preferences for medical treatment or care, due to physical deterioration or mental incapacity (9, 10). Moreover, since clinicians tend to focus on diagnosis, therapy and cure, the imminence of death is often not openly and timely acknowledged in patients with an advancing chronic illness (11, 12). A recent longitudinal study reported that end-of-life care was discussed between physicians and patients with terminal cancer in less than 20% of cases, and the frequency of these discussions only increased significantly in the last month of life (13). Consequently, patients often receive treatment aimed at prolonging life until a very late stage in their illness trajectory, with a considerable burden for the patient (14). Inadequately addressing the needs of the patients not only deteriorates the quality of patients' last phase of life (15), but also increases the risk of complicated grief in bereaved family members (16).

So far, studies have mostly explored the perspectives and experiences regarding factors that are important in end-of-life care of citizens and physicians (17-19), but the need to include the perspective of patients and their relatives has been acknowledged as well (4, 8, 20) . Studies investigating the needs and preferences of patients in their last phase of life have mostly included patients with cancer, and studied preferences on specific components of palliative care (21) . Little is known on patients' concerns, goals and sources of strength during their last phase of life (22). In addition, no studies have investigated these aspects within a context of diversity in diagnosis, culture, gender and age.

We expect that patients in the last phase of life consider dignity, respect, social relations, autonomy, symptoms and pain control as important. Although some of these themes may be universal, we

For peer review only - http://bmjopen.bmj.com/site/about/guidelines.xhtml

hypothesise that differences will exist in concerns, expectations and preferences based on gender, age, illness, care setting and culture.

The first aim of the iLIVE study is to provide in-depth understanding of the experiences, concerns, expectations and preferences of patients in the last phase of life and their relatives. The second aim is to assess variability in these concerns, expectations and preferences by culture, gender, and age, healthcare-related and socio-economic factors. The international character of the iLIVE study provides a framework for unprecedented international comparative insights. A better understanding of needs and outcomes in end-of-life care will thus contribute to the development and advancement of policies to support dignified dying in various cultures and settings.

For peer review only - http://bmjopen.bmj.com/site/about/guidelines.xhtml

METHODS AND ANALYSIS

Study design and setting

The iLIVE study is a prospective observational cohort study involving terminally ill patients in hospital and non-hospital sites in 11 participating countries: Argentina, Germany, Iceland, the Netherlands, New Zealand, Norway, Slovenia, Sweden, Switzerland, Spain, and the United Kingdom. Countries from three continents over the world were included in the study to ensure cultural diversity within the study population. Terminally ill patients will be followed until they die or for a maximum of six months after inclusion. Participating patients and one of their relatives will complete questionnaires about their experiences, concerns, expectations and preferences around dying and use of end-of-life care. This 4-year study started in September 2020 and is currently ongoing.

In order to have a diverse study population regarding clinical and sociodemographic characteristics, we will recruit participants from different types of clinical settings. Patients will be recruited in the 11 participating countries, from in total 20 hospitals (oncology, internal medicine, surgery, palliative care unit, medical physics, thoracic medicine and pulmonology departments), 7 specialized palliative care institutes, and 8 out-of-hospital settings (nursing homes).

Study population

In total, 2200 patients with a maximum estimated life expectancy of six months will be included, regardless of their diagnosis, gender, or place of residence (Table 1). Eligibility is assessed using a modified version of the Gold Standards Framework Proactive Identification Guidance (GSF-PIG) and the Supportive and Palliative Care Indicators Tool 2017 (SPICT) (20). The GSF-PIG starts with the “surprise question”, asking whether the physician would be surprised if a patient were to die within one year (23). For the present study, we adapted this question into whether the physician would be surprised if a patient were to die within six months. If the physician is uncertain about the surprise question, the patient is eligible when at least one SPICT indicator is present (24). SPICT is a tool to identify persons with poor or deteriorating health for assessment and care planning, using general indicators and clinical signs of life-limiting conditions (Supplemental Table 1). All physicians will be informed on how to apply the GSF-PIG and the SPICT tool to assess eligibility.

For peer review only - http://bmjopen.bmj.com/site/about/guidelines.xhtml

Participating patients are asked to identify a relative, for instance, a family member or friend. Relatives are eligible if they are 18 years of age or older. Patients and relatives need to be aware that the patient is unlikely to recover from his or her illness. The exclusion criteria for patients also apply to relatives.

Table 1. Inclusion and exclusion criteria for patients and relatives.

Inclusion criteria for patients	*Exclusion criteria for patients and relatives*
18 years of age or older	Unable to provide informed consent
Attending physician would not be surprised if the patient were to die within 6 months	Incapable of filling in questionnaires in the country's main language or in English
In case of uncertainty about surprise question: at least one SPICT indicator	
Awareness that recovery is unlikely	
Written informed consent to participate	
Inclusion criteria for relatives	
18 years of age or older	
Awareness that recovery of the patient is unlikely	
Written informed consent to participate	

Recruitment procedure

In the 11 countries, across all participating clinical sites, physicians are responsible for screening patients for eligibility. All consecutive patients admitted to a clinical ward or visiting an outpatient clinic will be screened for eligibility. Eligible patients are informed about the study by their attending physician or nurse, who provides them with an information leaflet. If patients agree to be informed about study participation, a researcher or research nurse from the local study team contacts them, answers their questions, and asks them if they consent to participate. If the patient consents, the researcher asks them to consider whether a close relative might also be willing to participate. After obtaining written informed consent from patients and, if applicable, relatives, they will be asked to fill in the baseline questionnaire.

In each country, five patients, five relatives and five healthcare professionals will be interviewed. Patients and relatives completing the questionnaire face-to-face will be asked whether they are interested in an additional in-depth interview. Patients and relatives completing the questionnaire

For peer review only - http://bmjopen.bmj.com/site/about/guidelines.xhtml

online or on paper (by post) will be approached by telephone. Patients and relatives who do not participate in the questionnaire study are also allowed to participate. If patients and/or relatives are eligible and interested, the researcher or research nurse approaches them to explain further procedures and to conduct the interview. They will have the option of participating in a face-to-face or skype interview.

Interviews will be conducted with healthcare professionals who are employed in the participating sites. Two criteria will be guiding the selection of healthcare professionals: (1) their work includes end-of-life care, and (2) they have several years of experience with end-of-life care. There will be variation in profession and work setting among participants. The healthcare professional will be contacted by telephone or email inviting them to take part in the study.

Measurements

The iLIVE cohort study includes several measurements (Table 2):

1) Questionnaires. Patients, relatives and attending physicians are asked to fill in questionnaires. Patients and relatives will complete questionnaires upon enrolment in the study (baseline assessment) and four weeks later (follow-up 1). For patients who die during the follow-up period of six months, relatives will also complete a questionnaire eight to ten weeks after the death of the patient (follow-up 2). Questionnaires for patients and relatives are administered on paper, online, or through telephone or face-to-face interviews. Physicians will complete a paper questionnaire at patient enrolment (baseline assessment) and after the death of a patient (follow-up 2).

   Completing the questionnaire will take approximately 30-45 minutes. In the online version of the questionnaire, participants are allowed to save their answers and continue at a later time point. The same is applicable to completing the paper version of the questionnaire and the face-to-face interview.

   Baseline assessment

   The baseline questionnaire for patients includes questions on their experiences, concerns, expectations and preferences around dying and end-of-life care. Questions also address health-related quality of life, symptoms, decision-making, social support, and about attitudes towards euthanasia. Finally, questions are asked about health economic aspects, such as patients'

For peer review only - http://bmjopen.bmj.com/site/about/guidelines.xhtml

employment status, use of healthcare and informal care needs. Relatives will also complete a questionnaire about their experiences, concerns, expectations and preferences around the last phase of life of the patient, their own health-related quality of life, their employment status and their provision of informal care. Attending physicians fill in a questionnaire about patients' diagnosis, co-morbidities, life expectancy, and their perspective on patients' current treatment aims. Where possible, validated measures that are commonly used to evaluate important aspects in end-of-life care are used to collect the data. (Table 2)

Follow-up 1

Four weeks after the baseline assessment, patients and relatives are asked to complete a follow-up questionnaire to assess changes as compared to baseline.

Follow-up 2

In case a participating patient dies, participating relatives are after eight to ten weeks asked to fill in a post-bereavement questionnaire, to assess their experience of the last days of life of the deceased patient, their appreciation of the quality of end-of-life care and family support, and their bereavement process. The physician or another healthcare staff member who attended the patient in the dying phase is also asked to complete a questionnaire to evaluate care in the dying phase. More specifically, questions will be asked on the place of death, symptoms and if they were treated, whether the patient and the family were informed that the patient was in the final stage of life, how long before death the patient lost the ability to express his/her will, and whether anyone was present at the time of death.

2) Medical file. Healthcare use in the patient's last week of life is assessed using a checklist. Items to be assessed include: place of care, medical complications, medication use, major medical and surgical interventions and care, goals of care statements, resuscitation policy and non-treatment decisions.

3) Qualitative interviews. More in-depth insight will be obtained in complementary personal interviews with patients, relatives and healthcare professionals. The same eligibility criteria apply as in the cohort study. The sample of interviewees will be controlled for age and gender per country, to allow a comparative analysis. The interviews will be semi-structured using a topic guide that is

For peer review only - http://bmjopen.bmj.com/site/about/guidelines.xhtml

based on Giger-Davidhizar-Haff's model for cultural assessment in end-of-life care (25), the ABCD model for effectively addressing and integrating cultural needs and issues in clinical care (26), and perception of disease questions (27).

During the interviews with patients, questions will be asked about their understanding of the illness, relationship with family, concerns, difficulties to discuss end-of-life topics, and decision-making. Comparable questions about these topics will be asked to relatives. Healthcare professionals will be asked questions about the care they aim to provide, collaboration with other professionals, communication with patients, decision-making, and values and beliefs when working with dying patients.

Table 2. Measurements among patients, relatives and physicians within the iLIVE project.

**I. Measured by questionnaire**	Measurement instrument
Patients	
- Concerns, expectations and preferences of patients around dying and end-of-life care	Self-developed questions adapted from the Serious Illness Conversation Guide (28) and the AEOLI questionnaire (29)
- Symptom load	Edmonton Symptom Assessment System (ESAS) (30)
- Health-related quality of life (HRQoL) and wellbeing	EORTC QLQ-C15-PAL quality of life question (31) & EuroQol 5 Dimension questionnaire (EQ-5D-5L) (32) ICECAP Supportive Care Measure (ICECAP-SCM) (33)
- Attitudes towards euthanasia[a]	10-item Euthanasia scale (34)
- Health and social care resource use, absenteeism from work	(Partial) Health Economics Questionnaire (HEQ)(35)
- Sociodemographic characteristics	Self-developed questions and HEQ
Relatives	
- Concerns, expectations and preferences around dying and end-of-life care	Self-developed questions inspired by the Serious Illness Conversation Guide and the AEOLI questionnaire
- Health-related quality of life (HRQoL)	EORTC QLQ-C15-PAL & EQ-5D-5L
- Well-being	ICECAP Close Person Questionnaire (ICECAP-CPM) (36)
- Informal care provision	iMTA Valuation of Informal Care Questionnaire (iVICQ)(37) and Informal Care Cost Assessment Questionnaire (CIIQ) (38)
- Attitudes towards euthanasia	10-item Euthanasia scale
- Bereavement	Hogan Grief Reaction Checklist (HGRC, despair and personal growth subscales) (39)
- Quality of care for dying patients	International questionnaire Care of the Dying Evaluation (iCODE) (40)
Physicians	

For peer review only - http://bmjopen.bmj.com/site/about/guidelines.xhtml

- Patients' diagnosis, co-morbidities and life expectancy, perspective on patients' treatment aims and functional status	Based on the SPICT-criteria and the Australian version of the Karnofsky Performance Status (41)
- Evaluation of care in the dying phase	Adapted and based on the Swedish Quality of Dying Registry (42)
**II. Obtained from medical files**	
- Use of medical interventions, medication and costs of medical care in the last week of life.	
- Patient survival	
**III. Obtained from qualitative interviews**	
- In-depth insights into experiences, concerns, expectations and preferences around dying and end-of-life care among patients, relatives and healthcare professionals.	

[a] In Norway and Iceland, one self-developed question will be used instead of the 10-item Euthanasia scale. No questions will be asked about euthanasia in Germany. Researchers from these countries were concerned that study participants would become anxious by these questions.

Translation of questionnaires

Where possible, published and validated versions of existing instruments in the languages of the participating countries will be used. Where necessary, instruments will be translated. An instrument that has been translated correctly is conceptually equivalent to the source instrument (43-45) and thereby enables collection and pooling data from various linguistic and cultural regions. Translations will be performed according to the standard proposed by the EORTC Quality of Life Group (46). The translation process will thus include two forward translations from English to the target language, development of a provisional consensus version, two backward translations, and a careful comparison with the original. This will be repeated iteratively until a satisfactory result is obtained. The original developers of the instruments will provide feedback during this process and approve the final translations. Self-developed questions will be developed in English and translated following the same standards. The final translations will also be tested as part of the study questionnaire pilot testing in each country.

Data management

This study will be conducted in accordance with the General Data Protection Regulation and national research ethics and privacy guidelines (47). One common data management system will be used to safely process and store data of all participating patients, relatives and physicians across clinical sites and countries. In some countries, participants can choose to directly enter data into this system; in that case, they consent to use of their e-mail address for communication purposes. In all other cases, data are entered anonymously by selected local research assistants, and a study number will be generated to link data of participants with a local communication database.

For peer review only

For peer review only - http://bmjopen.bmj.com/site/about/guidelines.xhtml

Sample size

The primary outcomes are measured at baseline and 4 weeks post-inclusion. It is expected that 30% of all patients who complete the baseline assessment will be lost to follow-up, due to death, significant deterioration of health, or other causes. In that case, 70% of patients who complete the baseline measurement will be able to complete the assessment after 4 weeks at follow-up 1. Further, it is expected that 80% of all patients who complete the baseline assessment can be followed until death, whereas the remaining 20% are expected to either survive until the end of the data collection period or become lost to follow-up. Regarding the relatives, it is expected that in case patients who complete the baseline assessment die during follow-up, half of the bereaved relatives (i.e. 40% of all baseline patients), will be willing to complete a post-bereavement questionnaire (follow-up 2). The total cohort would thus include 2200 patients (n=200 per country) at baseline, 1540 patients (n=140 per country) at follow-up assessment 1, and 880 bereaved relatives (n=80 per country). The number of 200 patients per country enables us to estimate proportions with 95% confidence intervals of approximately ±7%. The number of recruiting sites will vary from two to six per country.

No sample size estimation has been performed for the qualitative interviews since the aim is to explore and better understand the variety in experiences of patients, relatives, and physicians, rather than having a representative sample per country.

Analysis plan

Primary outcomes

The primary outcomes are experiences, concerns, expectations and preferences around dying and end-of-life care of patients in the last phase of life and their relatives, at baseline and after 4 weeks follow-up, and will be described in frequencies and narrative descriptions. The proportion of patients who have certain concerns, expectations and preferences will be described. Sub group analyses will be performed to assess cross-gender, cross-age and cross-cultural variety on experiences, concerns, expectations and preferences. Narrative descriptions will be translated into English and categorized into themes that will be identified within the data.

Descriptive statistics will be used to summarize baseline characteristics of the study participants (age, gender, education, diagnosis, comorbidities, religion, socioeconomic status, marital status, place of

For peer review only - http://bmjopen.bmj.com/site/about/guidelines.xhtml

residence, quality of life, symptom load) by country and site. Statistics on mean/median scores and variance will be presented where applicable. Associations with country and patient characteristics will be analysed in a multilevel modelling approach, taking account of clustering effects at country level. Both univariable and multivariable analyses will be performed. All statistical tests will be two-sided and considered significant if $p < 0.05$. Repeated measures analyses of variance will be conducted to assess the development of outcomes between baseline and 4 weeks follow-up. Multivariate Imputation by Chained Equations (MICE) will be used to handle missing data (48), as we expect that patients may not be able or want to fill in all questions in the questionnaire. MICE is known to be a flexible principled method of addressing missing data and can handle variables of varying types (e.g. continuous of binary). Quantitative analyses will be performed with SPSS 25.0 statistical software.

Secondary outcomes

Secondary outcomes for patients include symptom load, HRQoL and wellbeing, and attitudes towards physician-assistance in dying. Secondary outcomes for relatives include HRQoL, well-being, informal care provision, attitudes towards physician-assistance in dying and bereavement. The prevalence of these outcomes will be described in frequencies, mean/median scores and variance. Associations with country and patient characteristics will be analysed in a multilevel modelling approach, taking account of clustering effects at country level. Both univariable and multivariable analyses will be performed. Repeated measures analyses of variance will be conducted to assess the development of outcomes between baseline and 4 weeks follow-up. The relationship of the relative to the patient will be taken into account in multivariable models, in addition to the characteristics mentioned for the analysis of the primary outcome.

Health-economic analysis

The outcomes as assessed in this study allow inter alia for a comprehensive assessment of health resource utilization and costs for medication and care, as well as patients' and relatives' quality of life and well-being. The study therefore includes a cost-effectiveness analyses of interventions used in end-of-life care. In addition, a framework for the value assessment of palliative and end-of-life care will be developed (49).

For peer review only - http://bmjopen.bmj.com/site/about/guidelines.xhtml

Qualitative interviews

The interviews will be recorded and transcribed verbatim. Data will be thematically analysed in an iterative process on different levels: within each country, within three subgroups of countries and across all countries. The analysis will be focused on identifying experiences, concerns, expectations and preferences, as well as underlying values and norms. In addition, comparison of patients' perspectives will be explored by gender and age to gain a better understanding of differences in phenomena between subgroups. Data from the interviews will be imported into NVivo software for analysis.

Embedded intervention studies

The iLIVE project includes a number of studies that are embedded in the cohort study. The research protocols for these studies will be described elsewhere. A brief description is presented here.

iLIVE medication study

Discussion of appropriate medication to alleviate symptoms is one of the key clinical issues in improving care of dying patients (50). At the same time, potentially inappropriate medication is often continued until a very late stage in patients' illness trajectory (51). This concern will be addressed in the iLIVE Medication Study, in which a digital clinical tool, a so-called Clinical Decision Support System (CDSS), will be used to optimize medication management in the last phase of life. A previous version of this tool to guide physicians in medication prescription and de-prescription for residents of nursing homes was developed and tested in the Netherlands (52). In the iLIVE project, we developed an adapted version of this CDSS, the CDSS-OPTIMED, that supports physicians in optimizing their prescription of medications for patients with a limited life expectancy. The CDSS-OPTIMED will be evaluated in three countries participating in the iLIVE project (The Netherlands, Sweden and Switzerland).

iLIVE volunteer study

Volunteer services to support patients dying in hospitals, and their families, are relatively uncommon and empirical evidence of the usefulness of such services is scarce. This concern will be addressed in the iLIVE Volunteer Study, in which an international hospital palliative care volunteer training programme will be developed. This programme will underpin the implementation of palliative care volunteer services to support patients dying in hospital and their families, within five participating hospitals in five countries (the Netherlands, United Kingdom, Norway, Slovenia, and Spain). The iLIVE Volunteer Study will evaluate the implementation, use and experience of the iLIVE Volunteer Service.

For peer review only - http://bmjopen.bmj.com/site/about/guidelines.xhtml

Core Outcome Set for care of the dying

It is important to identify the most important outcomes for care of dying patients through the perspective of patients, family members, researchers, and health professionals. Despite a variety of available tools to assess different dimensions of palliative care, there is no consensus yet on which outcomes need to be measured in the last days of life. Therefore, this project will establish a Core Outcome Set (COS) for care of dying patients that includes valid, reliable and precise outcomes to enable international benchmarking, quality improvement and research in the last days of life (53). In each country, patients and relatives will be invited to participate during this process.

Patient and Public involvement

An Advisory Board (AB) will be established with research and clinical experts and representatives from all relevant stakeholder groups: current and future patients and their families, healthcare professionals, volunteers, policy makers, and researchers. The AB will engage in and advise on various aspects of the iLIVE project to ensure that the widest perspective on the process and outcomes can be realized.

In addition, in order to test the data collection for their acceptability and to maximize feasibility, we will pilot test the questionnaires in each country with 3-5 members from the target groups. Participants will be interviewed about their appreciation of the questionnaire following principles from cognitive interviewing techniques, which include open-ended questions as well specific probes (questions about potential problems). In case any modifications appear warranted, these will be discussed with the Project General Assembly. If relevant, modifications will be tested in additional patients.

For peer review only - http://bmjopen.bmj.com/site/about/guidelines.xhtml

ETHICS AND DISSEMINATION

The study will be conducted in accordance with national and international regulations and guidelines, including the Declaration of Helsinki (54), and the International Conference on Harmonization (ICH) guidance on Good Clinical Practice (GCP) (55). The study has been approved by Ethical Committees and institutional review boards (IRB's) in all participating countries. The following Ethical Commitees have approved the study:

- Regional Committee for Medical and Health Research Ethics South East D (35035), Norway.
- Komisija Republike Slovenije za Medicinsko etiko (0120-129/2020/3), Slovenia.
- Health Research Authority (HRA) and Health and Care Research Wales (HCRW) (272927), UK.
- Comité de Ética de la Investigación Provincial de Málaga. Hospital Regional Universitario de Malaga, Spain.
- Swedish Ethical Review Authority (2020-01956). Lund University, Norway.
- The National Bioethics Committee (VSN-20-129), Iceland.
- Ethics Commission of Cologne University, Faculty of Medicine (19-1456_1).
- Gesundheits-, Sozial und Integrationdirektion Kantonale Ethikkommission fur die Forschung (2020-02569), Switzerland.
- Medical research Ethics Committees United (MEC-U) (R20.004), The Netherlands.
- Dictamen del Comité de ética del instituto Lanari, University of Buenos Aires.

This study is registered in ClinicalTrials.gov (Trial Registration number NCT04271085). A Data Safety Monitoring Board (DSMB) has been established.

All potential participants to the study are provided with oral and written information about the study in the country's language. They will be given at least 72 hours (3 days) to consider participation and ask questions. All participants will be asked to provide written informed consent to confirm their willingness to participate in the study and for the data collection, storage and transfer of data according to established procedures.

We acknowledge the potential vulnerability of patients in the last phase of life and their relatives, and the risk of overburdening. Completing a questionnaire about concerns, preferences and expectations concerning the end of life can be uncomfortable. However, patients in the last phase of life and their caregivers have repeatedly been reported to appreciate being given the opportunity to participate in

For peer review only - http://bmjopen.bmj.com/site/about/guidelines.xhtml

research studies, even when they are close to death (56, 57). Participation in this study may nevertheless cause emotional burden for patients. Study participants will as a matter of principle be approached as people who are in principle fully capable of participating in research and whose experiences and concerns are important for healthcare professionals to learn from. If patients feel burdened by their participation, they are encouraged to indicate that on the questionnaire or to the researcher. Patients are also encouraged to discuss their issues with relatives or a healthcare professional.

The project results will be disseminated through the project website (www.iliveproject.eu), publications in scientific journals and at conferences. Within the project, there will be a working group focusing on enhancing the engagement of the community at large with the reality of death and dying. One of the aims is to actively promote societal debate and engagement with death and dying. This will be achieved by developing a detailed dissemination plan for efficient engagement of citizens, patients and families, healthcare professionals, volunteers and policymakers throughout the project, and effective dissemination of emerging outcomes.

For peer review only

For peer review only - http://bmjopen.bmj.com/site/about/guidelines.xhtml

DISCUSSION

The iLIVE study has several strengths. Going through the last phase of life is a complex personal experience, which is best understood while acknowledging the diverse and dynamic preferences of patients and their families. Due to the international nature of this project, we are able to investigate end-of-life experiences across different cultures and among groups varying by age, gender, illness and care setting. Further, we combine the perspectives of the most relevant stakeholders, that is, patients who are in the last phase of life and their relatives, as well as healthcare staff providing end-of-life care. Furthermore, patients and relatives will complete questionnaires at multiple time points, which enables us to analyse potential adaptations within subjects over time. Another strength relates to the post-bereavement assessment among relatives, which provides insights in the experience of care in the dying phase as well as the impact of these experiences on relatives' wellbeing and bereavement after the death of a patient. Lastly, the study population is relatively large which enables us to perform subgroup analyses.

We expect to encounter several challenges in this study. Recruiting patients in the last phase of life for research studies is often difficult. For instance, healthcare professionals or family members may be hesitant to provide researchers' access to incurably ill patients, due to concerns about burdening or distressing them, a phenomenon referred to as 'gatekeeping' (58). In many studies, this has led to considerably smaller study samples than desired. To minimize this risk, we have involved multiple clinical sites in almost all participating countries, planned for modest numbers of participants per site and applied conservative estimates of expected drop out. In addition, we will screen all potentially eligible patients and keep track of inclusion and exclusion numbers, as well as reasons for non-participation or exclusion.

Another challenge is that persons at the end of life may not be able to complete the follow-up questionnaire as they may become weaker over time. This will be monitored during the study and necessary actions will be taken in order to improve completion of the follow-up questionnaire.

In conclusion, the iLIVE project is aimed at increasing our understanding of the experience of dying in different settings and cultures around the world, and of the concerns, expectations, preferences and

For peer review only - http://bmjopen.bmj.com/site/about/guidelines.xhtml

needs of dying patients and their relatives. Such understanding is currently lacking, but key to the development of effective and efficient palliative and end-of-life care and public health policies.

For peer review only

For peer review only - http://bmjopen.bmj.com/site/about/guidelines.xhtml

Acknowledgements

-

Author contributions

The consortium has evolved from a successful collaboration of clinicians and researchers that developed through a joint interest and expertise in care of the dying to the "International Collaborative for Best Care for the Dying Person". AVDH, IJD, SA, MB[7], ME, JE, SE, CF, CJF, EG, AG, SIH, DFH, CH, TH, UL, MLMR, SM, BHR, MS, KRS, VS, JS, RM, KS, JS, VAT, HVDK, CCDVDR, LVZ, RV, SCZ contributed to the design of the study. BY wrote the first draft of the manuscript and revised with input from all authors. AVDH, IJD, SA, MB[7], ME, JE, SE, CF, CJF, EG, AG, SIH, DFH, CH, TH, UL, MLMR, SM, BHR, MS, KRS, VS, JS, RM, KS, JS, VAT, HVDK, CCDVDR, LVZ, RV, SCZ, MB[3], PBF, MB[6], AC, EECME, RH, GSI, MJ, HKC, NL, TMG, IRT critically reviewed the manuscript for important intellectual content. GGDS, GG, SM, VIV, EVM, MB[3], PBF, EECME, SIH, MJ, HKC, TMG, BHR, IRT, KS, VAT, EVB, SCZ provided feedback on the manuscript. All authors read and approved the final manuscript.

Ethics approval and consent to participate

The cohort study has been approved by Ethical Committees and the institutional review boards (IRB's) of participating institutes in all the countries. Physicians, (research) nurses and research staff will be involved in the recruitment of participants.

Consent to publish

Not applicable.

Competing interests

The authors declare that they have no competing interests.

Funding

This work is supported by the European Union's Horizon 2020 Research and Innovation Programme under Grant agreement no: 825731. The dissemination activities within the iLIVE project do not represent the opinion of the European Community and only reflect the opinion of the authors and/or the Consortium.

Data sharing statement

The study plans to share data per request and as overall study results.

For peer review only - http://bmjopen.bmj.com/site/about/guidelines.xhtml

REFERENCES

1. Heyland DK, Dodek P, Rocker G, Groll D, Gafni A, Pichora D, et al. What matters most in end-of-life care: perceptions of seriously ill patients and their family members. CMAJ : Canadian Medical Association journal = journal de l'Association medicale canadienne. 2006;174(5):627-33.
2. Chochinov HM, Hack T, Hassard T, Kristjanson LJ, McClement S, Harlos M. Dignity in the terminally ill: a cross-sectional, cohort study. Lancet. 2002;360(9350):2026-30.
3. Winter L. Patient values and preferences for end-of-life treatments: are values better predictors than a living will? Journal of palliative medicine. 2013;16(4):362-8.
4. Sandsdalen T, Hov R, Høye S, Rystedt I, Wilde-Larsson B. Patients' preferences in palliative care: a systematic mixed studies review. Palliative medicine. 2015;29(5):399-419.
5. Vig EK, Pearlman RA. Good and bad dying from the perspective of terminally ill men. Arch Intern Med. 2004;164(9):977-81.
6. Thomas C, Morris SM, Clark D. Place of death: preferences among cancer patients and their carers. Soc Sci Med. 2004;58(12):2431-44.
7. Hui D, dos Santos R, Chisholm GB, Bruera E. Symptom Expression in the Last Seven Days of Life Among Cancer Patients Admitted to Acute Palliative Care Units. J Pain Symptom Manage. 2015;50(4):488-94.
8. Kramer B, Auer C. Challenges to Providing End-of-Life Care to Low-Income Elders With Advanced Chronic Disease: Lessons Learned From a Model Program. The Gerontologist. 2005;45:651-60.
9. Silveira MJ, Kim SYH, Langa KM. Advance Directives and Outcomes of Surrogate Decision Making before Death. New England Journal of Medicine. 2010;362(13):1211-8.
10. Heyland DK, Barwich D, Pichora D, Dodek P, Lamontagne F, You JJ, et al. Failure to Engage Hospitalized Elderly Patients and Their Families in Advance Care Planning. JAMA Internal Medicine. 2013;173(9):778-87.
11. Emanuel EJ, Emanuel LL. The promise of a good death. The Lancet. 1998;351:SII21-SII9.
12. Lee DH. Approach to end of life care. The Ochsner journal. 2002;4(2):98-103.
13. Tang ST, Chen CH, Wen F-H, Chen J-S, Chang W-C, Hsieh C-H, et al. Accurate Prognostic Awareness Facilitates, Whereas Better Quality of Life and More Anxiety Symptoms Hinder End-of-Life Care Discussions: A Longitudinal Survey Study in Terminally Ill Cancer Patients' Last Six Months of Life. Journal of Pain and Symptom Management. 2018;55(4):1068-76.
14. Rochigneux P, Raoul JL, Beaussant Y, Aubry R, Goldwasser F, Tournigand C, et al. Use of chemotherapy near the end of life: what factors matter? Ann Oncol. 2017;28(4):809-17.
15. Raijmakers NJH, Zijlstra M, Van Roij J, Husson O, Oerlemans S, Van De Poll-Franse LV. Health-related quality of life among cancer patients in their last year of life: results from the PROFILES registry. Supportive Care in Cancer. 2018;26(10):3397-404.
16. Jonasson JM, Hauksdóttir A, Valdimarsdóttir U, Fürst CJ, Onelöv E, Steineck G. Unrelieved symptoms of female cancer patients during their last months of life and long-term psychological morbidity in their widowers: A nationwide population-based study. European Journal of Cancer. 2009;45(10):1839-45.
17. Wong A, Zhukovsky DS. Chapter 3 - Patient Experience and End-of-Life Care: A Discussion and Analysis of Four Patients. In: Gallagher C, Ewer M, editors. Ethical Challenges in Oncology. Boston: Academic Press; 2017. p. 41-57.
18. Black A, McGlinchey T, Gambles M, Ellershaw J, Mayland CR. The 'lived experience' of palliative care patients in one acute hospital setting – a qualitative study. BMC Palliative Care. 2018;17(1):91.

For peer review only - http://bmjopen.bmj.com/site/about/guidelines.xhtml

19. Sampson C, Finlay I, Byrne A, Snow V, Nelson A. The practice of palliative care from the perspective of patients and carers. BMJ Supportive & Palliative Care. 2014;4(3):291.
20. www.goldstandardsframework.org.uk/PIG . (Accessed 6 October 2020)
21. Davies E, Higginson IJ, World Health O. The solid facts: palliative care. Copenhagen: WHO Regional Office for Europe; 2004.
22. Vig EK, Davenport NA, Pearlman RA. Good deaths, bad deaths, and preferences for the end of life: a qualitative study of geriatric outpatients. Journal of the American Geriatrics Society. 2002;50(9):1541-8.
23. White N, Kupeli N, Vickerstaff V, Stone P. How accurate is the 'Surprise Question' at identifying patients at the end of life? A systematic review and meta-analysis. BMC Medicine. 2017;15(1):139.
24. Highet G, Crawford D, Murray SA, Boyd K. Development and evaluation of the Supportive and Palliative Care Indicators Tool (SPICT): a mixed-methods study. BMJ Support Palliat Care. 2014;4(3):285-90.
25. Giger JN, Davidhizar R. The Giger and Davidhizar Transcultural Assessment Model. J Transcult Nurs. 2002;13(3):185-8; discussion 200-1.
26. Srivastava R. The ABC (and DE) of cultural competence in clinical care. Ethnicity and Inequalities in Health and Social Care. 2008;1(1):27-33.
27. Price A, Goodwin L, Rayner L, Shaw E, Hansford P, Sykes N, et al. Illness perceptions, adjustment to illness, and depression in a palliative care population. Journal of pain and symptom management. 2012;43(5):819-32.
28. Bernacki R, Hutchings M, Vick J, Smith G, Paladino J, Lipsitz S, et al. Development of the Serious Illness Care Program: a randomised controlled trial of a palliative care communication intervention. BMJ Open. 2015;5(10):e009032.
29. Catt S, Blanchard M, Addington-Hall J, Zis M, Blizard B, King M. The development of a questionnaire to assess the attitudes of older people to end-of-life issues (AEOLI). Palliative medicine. 2005;19(5):397-401.
30. Bruera E, Kuehn N, Miller MJ, Selmser P, Macmillan K. The Edmonton Symptom Assessment System (ESAS): a simple method for the assessment of palliative care patients. J Palliat Care. 1991;7(2):6-9.
31. Groenvold M, Petersen MA, Aaronson NK, Arraras JI, Blazeby JM, Bottomley A, et al. The development of the EORTC QLQ-C15-PAL: a shortened questionnaire for cancer patients in palliative care. Eur J Cancer. 2006;42(1):55-64.
32. Euroqol. https://euroqol.org/eq-5d-instruments/eq-5d-5l-about/
33. Bailey C, Kinghorn P, Orlando R, Armour K, Perry R, Jones L, et al. 'The ICECAP-SCM tells you more about what I'm going through': A think-aloud study measuring quality of life among patients receiving supportive and palliative care. Palliative medicine. 2016;30(7):642-52.
34. Wasserman J, Clair JM, Ritchey FJ. A scale to assess attitudes toward euthanasia. OMEGA-Journal of Death and Dying. 2005;51(3):229-37.
35. Simon J, Mayer S. HEQ (Health Economics Questionnaire). Zenodo. 2016.
36. Canaway A, Al-Janabi H, Kinghorn P, Bailey C, Coast J. Development of a measure (ICECAP-Close Person Measure) through qualitative methods to capture the benefits of end-of-life care to those close to the dying for use in economic evaluation. Palliative medicine. 2017;31(1):53-62.
37. Hoefman RJ, Van Exel NJA, Brouwer WBF. iMTA valuation of informal care questionnaire MANUAL. 2013.
38. Landfeldt E, Zethraeus N, Lindgren P. Standardized questionnaire for the measurement, valuation, and estimation of costs of informal care based on the opportunity cost and proxy good method. Applied health economics and health policy. 2019;17(1):15-24.

For peer review only - http://bmjopen.bmj.com/site/about/guidelines.xhtml

39. S. Hogan DBGLASN. Development and validation of the Hogan grief reaction checklist. Death studies. 2001;25(1):1-32.
40. Mayland CR, Gerlach C, Sigurdardottir K, Hansen MIT, Leppert W, Stachowiak A, et al. Assessing quality of care for the dying from the bereaved relatives' perspective: Using pre-testing survey methods across seven countries to develop an international outcome measure. Palliat Med. 2019;33(3):357-68.
41. Abernethy AP, Shelby-James T, Fazekas BS, Woods D, Currow DC. The Australia-modified Karnofsky Performance Status (AKPS) scale: a revised scale for contemporary palliative care clinical practice [ISRCTN81117481]. BMC Palliative Care. 2005;4(1):7.
42. Martinsson L, Heedman PA, Lundström S, Fransson G, Axelsson B. Validation study of an end-of-life questionnaire from the Swedish Register of Palliative Care. Acta Oncol. 2011;50(5):642-7.
43. Edition F, Kuliś D, Bottomley A, Velikova G, Greimel E, Koller M. Eortc Quality of Life Group Translation Procedure.
44. Sousa VD, Rojjanasrirat W. Translation, adaptation and validation of instruments or scales for use in cross-cultural health care research: a clear and user-friendly guideline. Journal of evaluation in clinical practice. 2011;17(2):268-74.
45. Beaton DE, Bombardier C, Guillemin F, Ferraz MB. Guidelines for the process of cross-cultural adaptation of self-report measures. Spine. 2000;25(24):3186-91.
46. http://groups.eortc.be/qol/sites/default/files/img/newsletter/translation_manual_2017.pdf
47. https://www.eugdpr.org/.
48. Azur MJ, Stuart EA, Frangakis C, Leaf PJ. Multiple imputation by chained equations: what is it and how does it work? Int J Methods Psychiatr Res. 2011;20(1):40-9.
49. Fischer C, Chwala E, Simon J. Methodological aspects of economic evaluations conducted in the palliative or end of life care settings: a systematic review protocol. BMJ Open. 2020;10(5):e035760.
50. Todd A, Holmes HM. Recommendations to support deprescribing medications late in life. International journal of clinical pharmacy. 2015;37(5):678-81.
51. Arevalo JJ, Geijteman ECT, Huisman BAA, Dees MK, Zuurmond WWA, van Zuylen L, et al. Medication Use in the Last Days of Life in Hospital, Hospice, and Home Settings in the Netherlands. J Palliat Med. 2018;21(2):149-55.
52. Mestres Gonzalvo C, de Wit HAJM, van Oijen BPC, Hurkens KPGM, Janknegt R, Schols JMGA, et al. Supporting clinical rules engine in the adjustment of medication (SCREAM): protocol of a multicentre, prospective, randomised study. BMC Geriatr. 2017;17(1):35-.
53. Zambrano SC, Haugen DF, van der Heide A, Tripodoro VA, Ellershaw J, Fürst CJ, et al. Development of an international Core Outcome Set (COS) for best care for the dying person: study protocol. BMC Palliat Care. 2020;19(1):184.
54. World Medical A. World Medical Association Declaration of Helsinki: Ethical Principles for Medical Research Involving Human Subjects. Jama. 2013;310(20):2191-4.
55. International Conference on Harmonisation (ICH) Good Clinical Practice Standards (E6 document). 1996.
56. Gysels MH, Evans C, Higginson IJ. Patient, caregiver, health professional and researcher views and experiences of participating in research at the end of life: a critical interpretive synthesis of the literature. BMC Med Res Methodol. 2012;12:123.
57. Higginson IJ, Evans CJ, Grande G, Preston N, Morgan M, McCrone P, et al. Evaluating complex interventions in end of life care: the MORECare statement on good practice generated by a synthesis of transparent expert consultations and systematic reviews. BMC medicine. 2013;11(1):111.
58. Kars MC, van Thiel GJ, van der Graaf R, Moors M, de Graeff A, van Delden JJ. A systematic review of reasons for gatekeeping in palliative care research. Palliat Med. 2016;30(6):533-48.

For peer review only - http://bmjopen.bmj.com/site/about/guidelines.xhtml

1

For peer review only

For peer review only - http://bmjopen.bmj.com/site/about/guidelines.xhtml

Supplementary Table 1. The Supportive and Palliative Care Indicators Tool (SPICT).

General SPICT indicators:
• Unplanned hospital admission.<br>• Performance status is poor or deteriorating, with limited reversibility (e.g. stays in bed or in a chair for more than half the day).<br>• Depends on others for care due to increasing physical and/or mental health problems; person's carer needs more help and support.<br>• Progressive weight loss; remains underweight; low muscle mass.<br>• Persistent symptoms despite optimal treatment of underlying condition(s).<br>• Person (or family) asks for palliative care; chooses to reduce, stop or not have treatment; or wishes to focus on quality of life.
**Disease-specific SPICT indicators:**
Cancer:<br>• Functional ability deteriorating due to progressive cancer.<br>• Too frail for cancer treatment or treatment is for symptom control.
Neurological disease:<br>• Progressive deterioration in physical and/or cognitive function despite optimal therapy.<br>• Speech problems with increasing difficulty communicating and/or progressive difficulty with swallowing.<br>• Recurrent aspiration pneumonia; breathless or respiratory failure.<br>• Persistent paralysis after stroke with significant loss of function and ongoing disability.
Heart/vascular disease:<br>• Heart failure or extensive, untreatable coronary artery disease; with breathlessness or chest pain at rest or on minimal effort.<br>• Severe, inoperable peripheral vascular disease.
Respiratory disease:<br>• Severe, chronic lung disease; with breathlessness at rest or on minimal effort between exacerbations.<br>• Persistent hypoxia needing long-term oxygen therapy.<br>• Has needed ventilation for respiratory failure or ventilation is contraindicated.
Kidney disease:<br>• Stage 4 or 5 chronic kidney disease (eGFR < 30ml/min) with deteriorating health.<br>• Kidney failure complicating other life limiting conditions or treatments.<br>• Stopping or not starting dialysis.
Liver disease:<br>• Cirrhosis with one or more complications in the past year: diuretic resistant ascites; hepatic encephalopathy; hepatorenal syndrome; bacterial peritonitis; or recurrent variceal bleeds.<br>• Liver transplant is not possible.
Dementia/ frailty[1]:<br>• Unable to dress, walk or eat without help.<br>• Eating and drinking less, difficulty with swallowing.<br>• Urinary and faecal incontinence.<br>• Not able to communicate by speaking; little social interaction.<br>• Frequent falls; fractured femur.<br>• Recurrent febrile episodes or infections, aspiration pneumonia.
Other conditions:<br>• Deteriorating and at risk of dying with other conditions or complications that are not reversible; any treatment available will have a poor outcome.

[1] If a patient with mild cognitive impairment is considered eligible, the physician is requested to assess this patient's capacity using a locally available validated capacity assessment instrument.

For peer review only

Supplementary Table 2. Measurement instruments and their scale scores used in the iLIVE study.

Topic	Measurement instrument	Scale scores
Patients		
- Concerns, expectations and preferences of patients around dying and end-of-life care	- Self-developed questions adapted from the Serious Illness Conversation Guide (1)<br>- AEOLI questionnaire (2)	Not applicable<br>Strongly disagree – disagree- neither agree nor disagree – agree- strongly agree – don't know
- Symptom load	Edmonton Symptom Assessment System (ESAS) (3)	0 (no symptom) – 10 (worst possible symptom)
- Health-related quality of life (HRQoL) and wellbeing	EORTC QLQ-C15-PAL quality of life question (4)<br>EuroQol 5 Dimension questionnaire (EQ-5D-5L) (5)<br>ICECAP Supportive Care Measure (ICECAP-SCM) (6)	0 (worst health) – 100 (best health)<br>Questions 1-3: no problems – slight problems- moderate problems – severe problems - unable<br>Questions 4 (pain) & 5 (anxious): no(t) – slight – moderate – severe – extreme(ly)<br>Most of the time –some of the time – only a little of the time - never
- Attitudes towards euthanasia[a]	10-item Euthanasia scale (7)	Strongly disagree – disagree- neither agree nor disagree – agree- strongly agree – don't know
- Health and social care resource use, absenteeism from work	(Partial) Health Economics Questionnaire (HEQ)(8)	Not applicable
- Sociodemographic characteristics	Self-developed questions and HEQ	Not applicable
Relatives		
- Concerns, expectations and preferences around dying and end-of-life care	Self-developed questions inspired by the Serious Illness Conversation Guide and the AEOLI questionnaire	Not applicable

For peer review only

- Health-related quality of life (HRQoL)	EORTC QLQ-C15-PAL<br><br>EQ-5D-5L	0 (worst health) – 100 (best health)<br><br>Questions 1-3: no problems – slight problems- moderate problems – severe problems - unable<br><br>Questions 4 (pain) & 5 (anxious): no(t) – slight – moderate – severe – extreme(ly)
- Well-being	ICECAP Close Person Questionnaire (ICECAP-CPM) (9)	Question 1 -2 : all of the time- most- some- a little- non<br><br>Question 3-6: fully able – mostly able- mostly unable –completely unable
- Informal care provision	iMTA Valuation of Informal Care Questionnaire (iVICQ)(10) and Informal Care Cost Assessment Questionnaire (CIIQ) (11)	Not applicable
- Attitudes towards euthanasia	10-item Euthanasia scale	Strongly disagree – disagree- neither agree nor disagree – agree- strongly agree – don't know
- Bereavement	Hogan Grief Reaction Checklist (HGRC, despair and personal growth subscales) (12)	1= Does not describe me at all<br>2 = Does not quite describe me<br>3 = Describes me fairly well<br>4 = Describes me well<br>5 = Describes me very well
- Quality of care for dying patients	International questionnaire Care of the Dying Evaluation (iCODE) (13)	Various scales
Physicians		
- Patients' diagnosis, co-morbidities and life expectancy, perspective on	Based on the SPICT-criteria and the Australian version of the Karnofsky Performance Status (14)	Not applicable

For peer review only

	patients' treatment aims and functional status		
-	Evaluation of care in the dying phase	Adapted and based on the Swedish Quality of Dying Registry (15)	Various scales

For peer review only

References

1. Bernacki R, Hutchings M, Vick J, Smith G, Paladino J, Lipsitz S, et al. Development of the Serious Illness Care Program: a randomised controlled trial of a palliative care communication intervention. BMJ Open. 2015;5(10):e009032.
2. Catt S, Blanchard M, Addington-Hall J, Zis M, Blizard B, King M. The development of a questionnaire to assess the attitudes of older people to end-of-life issues (AEOLI). Palliative medicine. 2005;19(5):397-401.
3. Bruera E, Kuehn N, Miller MJ, Selmser P, Macmillan K. The Edmonton Symptom Assessment System (ESAS): a simple method for the assessment of palliative care patients. J Palliat Care. 1991;7(2):6-9.
4. Groenvold M, Petersen MA, Aaronson NK, Arraras JI, Blazeby JM, Bottomley A, et al. The development of the EORTC QLQ-C15-PAL: a shortened questionnaire for cancer patients in palliative care. Eur J Cancer. 2006;42(1):55-64.
5. Euroqol. https://euroqol.org/eq-5d-instruments/eq-5d-5l-about/
6. Bailey C, Kinghorn P, Orlando R, Armour K, Perry R, Jones L, et al. 'The ICECAP-SCM tells you more about what I'm going through': A think-aloud study measuring quality of life among patients receiving supportive and palliative care. Palliative medicine. 2016;30(7):642-52.
7. Wasserman J, Clair JM, Ritchey FJ. A scale to assess attitudes toward euthanasia. OMEGA-Journal of Death and Dying. 2005;51(3):229-37.
8. Simon J, Mayer S. HEQ (Health Economics Questionnaire). Zenodo. 2016.
9. Canaway A, Al-Janabi H, Kinghorn P, Bailey C, Coast J. Development of a measure (ICECAP-Close Person Measure) through qualitative methods to capture the benefits of end-of-life care to those close to the dying for use in economic evaluation. Palliative medicine. 2017;31(1):53-62.
10. Hoefman RJ, Van Exel NJA, Brouwer WBF. iMTA valuation of informal care questionnaire MANUAL. 2013.
11. Landfeldt E, Zethraeus N, Lindgren P. Standardized questionnaire for the measurement, valuation, and estimation of costs of informal care based on the opportunity cost and proxy good method. Applied health economics and health policy. 2019;17(1):15-24.
12. S. Hogan DBGLASN. Development and validation of the Hogan grief reaction checklist. Death studies. 2001;25(1):1-32.
13. Mayland CR, Gerlach C, Sigurdardottir K, Hansen MIT, Leppert W, Stachowiak A, et al. Assessing quality of care for the dying from the bereaved relatives' perspective: Using pre-testing survey methods across seven countries to develop an international outcome measure. Palliat Med. 2019;33(3):357-68.
14. Abernethy AP, Shelby-James T, Fazekas BS, Woods D, Currow DC. The Australia-modified Karnofsky Performance Status (AKPS) scale: a revised scale for contemporary palliative care clinical practice [ISRCTN81117481]. BMC Palliative Care. 2005;4(1):7.
15. Martinsson L, Heedman PA, Lundström S, Fransson G, Axelsson B. Validation study of an end-of-life questionnaire from the Swedish Register of Palliative Care. Acta Oncol. 2011;50(5):642-7.

For peer review only

STROBE 2007 (v4) Statement—Checklist of items that should be included in reports of *cohort studies*

Section/Topic	Item #	Recommendation	Reported on page #
**Title and abstract**	1	(*a*) Indicate the study's design with a commonly used term in the title or the abstract	0
		(*b*) Provide in the abstract an informative and balanced summary of what was done and what was found	1
**Introduction**			
Background/rationale	2	Explain the scientific background and rationale for the investigation being reported	4
Objectives	3	State specific objectives, including any prespecified hypotheses	4
**Methods**			
Study design	4	Present key elements of study design early in the paper	6
Setting	5	Describe the setting, locations, and relevant dates, including periods of recruitment, exposure, follow-up, and data collection	6
Participants	6	(*a*) Give the eligibility criteria, and the sources and methods of selection of participants. Describe methods of follow-up	6/7
		(*b*) For matched studies, give matching criteria and number of exposed and unexposed	na
Variables	7	Clearly define all outcomes, exposures, predictors, potential confounders, and effect modifiers. Give diagnostic criteria, if applicable	7-9
Data sources/ measurement	8*	For each variable of interest, give sources of data and details of methods of assessment (measurement). Describe comparability of assessment methods if there is more than one group	*8-9*
Bias	9	Describe any efforts to address potential sources of bias	na
Study size	10	Explain how the study size was arrived at	10
Quantitative variables	11	Explain how quantitative variables were handled in the analyses. If applicable, describe which groupings were chosen and why	na
Statistical methods	12	(*a*) Describe all statistical methods, including those used to control for confounding	11
		(*b*) Describe any methods used to examine subgroups and interactions	11
		(*c*) Explain how missing data were addressed	11
		(*d*) If applicable, explain how loss to follow-up was addressed	11
		(*e*) Describe any sensitivity analyses	na
**Results**			

For peer review only

Participants	13*	(a) Report numbers of individuals at each stage of study—eg numbers potentially eligible, examined for eligibility, confirmed eligible, included in the study, completing follow-up, and analysed	na
		(b) Give reasons for non-participation at each stage	na
		(c) Consider use of a flow diagram	na
Descriptive data	14*	(a) Give characteristics of study participants (eg demographic, clinical, social) and information on exposures and potential confounders	na
		(b) Indicate number of participants with missing data for each variable of interest	na
		(c) Summarise follow-up time (eg, average and total amount)	na
Outcome data	15*	Report numbers of outcome events or summary measures over time	na
Main results	16	(*a*) Give unadjusted estimates and, if applicable, confounder-adjusted estimates and their precision (eg, 95% confidence interval). Make clear which confounders were adjusted for and why they were included	na
		(*b*) Report category boundaries when continuous variables were categorized	na
		(*c*) If relevant, consider translating estimates of relative risk into absolute risk for a meaningful time period	na
Other analyses	17	Report other analyses done—eg analyses of subgroups and interactions, and sensitivity analyses	na
**Discussion**			
Key results	18	Summarise key results with reference to study objectives	na
**Limitations**			
Interpretation	20	Give a cautious overall interpretation of results considering objectives, limitations, multiplicity of analyses, results from similar studies, and other relevant evidence	na
Generalisability	21	Discuss the generalisability (external validity) of the study results	14
**Other information**			
Funding	22	Give the source of funding and the role of the funders for the present study and, if applicable, for the original study on which the present article is based	16

*Give information separately for cases and controls in case-control studies and, if applicable, for exposed and unexposed groups in cohort and cross-sectional studies.

Note: An Explanation and Elaboration article discusses each checklist item and gives methodological background and published examples of transparent reporting. The STROBE checklist is best used in conjunction with this article (freely available on the Web sites of PLoS Medicine at http://www.plosmedicine.org/, Annals of Internal Medicine at http://www.annals.org/, and Epidemiology at http://www.epidem.com/). Information on the STROBE Initiative is available at www.strobe-statement.org.

For peer review only
